# Reduced CCR5 Expression and Immune Quiescence in Black South African HIV-1 Controllers

**DOI:** 10.3389/fimmu.2021.781263

**Published:** 2021-12-20

**Authors:** Anabela C. P. Picton, Maria Paximadis, Gemma W. Koor, Avani Bharuthram, Sharon Shalekoff, Ria Lassauniere, Prudence Ive, Caroline T. Tiemessen

**Affiliations:** ^1^ Centre for HIV and STIs, National Institute for Communicable Diseases, Johannesburg, South Africa; ^2^ Department of Virology, School of Pathology, Faculty of Health Sciences, University of the Witwatersrand, Johannesburg, South Africa; ^3^ Virus Research and Development Laboratory, Department of Virus and Microbiological Special Diagnostics, Statens Serum Institut, Copenhagen, Denmark; ^4^ Clinical HIV Research Unit, Department of Internal Medicine, School of Clinical Medicine, Faculty of Health Sciences, University of the Witwatersrand, Johannesburg, South Africa

**Keywords:** HIV-1 control, CCR5 expression, immune activation, CCR5 ligands, IL-10

## Abstract

Unique Individuals who exhibit either suppressive HIV-1 control, or the ability to maintain low viral load set-points and preserve their CD4+ T cell counts for extended time periods in the absence of antiretroviral therapy, are broadly termed HIV-1 controllers. We assessed the extent to which black South African controllers (n=9), differ from uninfected healthy controls (HCs, n=22) in terms of lymphocyte and monocyte CCR5 expression (density and frequency of CCR5-expressing cells), immune activation as well as peripheral blood mononuclear cell (PBMC) mitogen-induced chemokine/cytokine production. In addition, relative CD4+ T cell CCR5 mRNA expression was assessed in a larger group of controllers (n=20) compared to HCs (n=10) and HIV-1 progressors (n=12). Despite controllers having significantly higher frequencies of activated CD4+ and CD8+ T cells (HLA-DR+) compared to HCs, CCR5 density was significantly lower in these T cell populations (*P*=0.039 and *P*=0.064, respectively). This lower CCR5 density was largely attributable to controllers with higher VLs (>400 RNA copies/ml). Significantly lower CD4+ T cell CCR5 density in controllers was maintained (*P*=0.036) when HCs (n=12) and controllers (n=9) were matched for age. CD4+ T cell CCR5 mRNA expression was significantly less in controllers compared to HCs (*P*=0.007) and progressors (*P*=0.002), whereas HCs and progressors were similar (*P*=0.223). The levels of soluble CD14 in plasma did not differ between controllers and HCs, suggesting no demonstrable monocyte activation. While controllers had lower monocyte CCR5 density compared to the HCs (*P*=0.02), significance was lost when groups were age-matched (*P*=0.804). However, when groups were matched for both CCR5 promoter haplotype and age (n=6 for both) reduced CCR5 density on monocytes in controllers relative to HCs was highly significant (*P*=0.009). Phytohemagglutinin-stimulated PBMCs from the controllers produced significantly less CCL3 (*P*=0.029), CCL4 (*P*=0.008) and IL-10 (P=0.028) compared to the HCs, which was largely attributable to the controllers with lower VLs (<400 RNA copies/ml). Our findings support a hypothesis of an inherent (genetic) predisposition to lower CCR5 expression in individuals who naturally control HIV-1, as has been suggested for Caucasian controllers, and thus, likely involves a mechanism shared between ethnically divergent population groups.

## Introduction

People living with HIV (PLWH) who are able to naturally control HIV infection are likely to possess genetic or immunological attributes that could provide important insights for the development of therapeutic agents and to inform HIV cure strategies and vaccine design. However, despite the high burden of disease (1), studies assessing protective mechanisms in sub-Saharan PLWH who exhibit good control of HIV-1 infection/disease are more limited.

HIV-1-infected progressors and non-progressors have been described to differ in their host gene complement, viral strains as well as their immunological responses (2). Although non-progression in HIV-infected individuals has been attributed to infection with attenuated viruses in a minority of reports (3, 4), other studies report non-progression in individuals infected with fully replication-competent HIV-1 viruses (5–7), lending support to the idea that host factors play a large role in delayed disease progression. The role of CCR5 coreceptor density in the susceptibility of an individual to HIV-1 has been well established. The *CCR5Δ32* allele has been reported as over-represented within groups of patients infected with HIV-1 who progress to disease at slower than normal rates (8–10). This deletion results in truncation of the expressed protein and prevents the expression of CCR5 on the cell surface (11). Furthermore, high CCR5 expression on CD4+ T cells associates with high viral loads (VLs) and accelerated disease progression (12, 13). However, although the *CCR5Δ32* allele is virtually absent in sub-Saharan populations (14, 15), *CCR5* promoter haplotypes have been demonstrated to affect CCR5 surface expression in cohorts of South African individuals (16, 17). This has been demonstrated in individuals with and without HIV-1 infection.

It is well established that immune activation is a hallmark of pathogenic HIV-1 infection. Immune activation levels serve as the best predictors of disease progression to AIDS and death, independently of HIV-1 VL (18–20). Several lines of evidence point to CCR5 functioning as a molecule that enhances T cell activation. Antibody-mediated blockade of the CCR5-CCR5 ligand axis has been demonstrated to result in lower expression of IL-2, IFNγ and CD25 - molecules that serve as markers of cellular activation (21–23). CCR5 expression influences IL-2 and CD25 expression through regulation of the intracellular levels of NFAT (nuclear factor of activated T cells) (23). During T cell stimulation, CCR5 molecules are sequestered to the immunological synapse where they are stimulated and deliver costimulatory signals (24, 25). In contrast to CXCR4-utilising strains, those that utilise CCR5 enhance CD4+ T cell activation, thus favouring HIV replication and spread (26). In addition, the function of CCR5 as a costimulatory molecule is dependent on the level of CCR5 cell surface expression. CCR5 density correlates with, and is predictive of, the immune activation levels of HIV-1-infected individuals independently of VL (27). CCR5 density on naïve CD4+ T cells is unaffected by neither the initiation of antiretroviral therapy (ART) nor ART treatment interruption, despite the respective decrease and increase in the proportion of activated CD8+ T cells (CD38^hi^) - i.e., the baseline level of CCR5 density is a determinant of the intensity of immune activation (27). Gornalusse et al. (28) showed an inverse correlation between the DNA methylation status of the *CCR5 cis*-regulatory regions and CCR5 levels on T cells, and that T cell activation induced demethylation of these regions, leading to upregulation of CCR5 expression. Furthermore, they showed that polymorphisms in *CCR5 cis*-regulatory regions that associated with increased and decreased HIV/AIDS susceptibility were also associated with increased and decreased sensitivity to activation-induced demethylation, respectively (28).

We previously reported that the cell surface density of CCR5 and proportions of CCR5-expressing cells differ significantly between white and black South African individuals who are HIV-1 uninfected (29). Generally, white individuals displayed higher CCR5 cell density, whereas black individuals had higher proportions of CCR5-expressing cells, which correlated positively with the proportions of activated cells (29). To our knowledge, no studies have directly assessed the role of CCR5 expression on natural control of HIV-1 in a sub-Saharan population.

## Materials and Methods

### Study Cohorts

The majority of the work reported in this study (CCR5 expression and cytokine production) has been conducted on a small group of well characterized black South African HIV-1 controllers (n=9) and a group of black South African healthy control donors (n=22) - termed cohort 1. The HIV-1-infected controllers in this cohort comprised 9 black South African individuals infected with HIV-1 with long-term follow-up that had been prospectively recruited. These individuals were a mixture of those with suppressive viral control (i.e. elite controllers) or with low viral set points (viraemic controllers and/or long-term non-progressors). Criteria for selection were the sustained control of disease in the absence of antiretroviral treatment (ART) for a period of ≥6 years and/or consistently high CD4+ T cell counts. This group comprised six females and three males and had a median age of 38 years (range: 32-54 years) at the time at which the experiments were conducted (**Table 1**). Among the group of controllers, two individuals (TG11 and Pru1) met the criteria of elite controllers i.e., patients with plasma HIV RNA levels of <50 copies/ml (30). At the time of this study, the median number of years of infection without treatment for this cohort was 9 years (range: 6-14), and subsequent to this study (last recorded data), the median number of years of infection without treatment was 14 years (range: 7-20; **Table 1**).

**Table 1:** Characteristics of HIV-1 controllers (cohort 1).

Patient ID	Age (years)	Gender	Viral load (RNA copies/ml)	CD4+ T cell count (cells/μl)	Time since diagnosis for current study (years)	Time since diagnosis without ARVs^1^ (years)	*CCR5* genotype
TG1	38	M	6 070	334	9	11	HHA/HHF*2
TG2	47	F	5 780	400	6	7	HHA/HHF*2
**TG4**	35	M	183	910	9	14	HHA/HHA
TG9	46	F	<400	327	9	12	HHE/HHF*2
**TG11**	32	F	<40	693	7	12	HHA/HHC
**Pru1**	54	F	<40	>2000	14	20	HHC/HHC
**Pru2**	43	M	1 155	637	14	20	HHF*1/HHG*1
**Pru3**	36	F	1 410	775	11	16	HHA/HHC
Pru4	38	F	124	379	13	19	HHC/HHD

^1^The time since diagnosis in the absence of antiretroviral drugs (ARVs) up to the latest follow-up point or up to the initiation of ARVs – experiments in current study were thus performed on samples collected at earlier time points.

Bold patient IDs indicate the 5 controllers that were included in the CCR5 mRNA expression (CD4+ T cell) experiments (cohort 2)

The group of 22 healthy black South African individuals, without HIV-1 infection (HCs), has been previously described (29). The age and gender of the HC participants are listed in **Supplementary Table 1**. Although attempts were made to age and sex match the HIV-1 controllers and the HCs, the HCs had a trend of lower age compared to the controllers (medians: 32.5 vs. 38 years, respectively; *P*=0.05). There was no difference in the male:female ratio between the two groups (*P*=1).

A second cohort (cohort 2) was used to compare CCR5 mRNA expression in CD4+ T cells between a larger group of black South African HIV-1 controllers (n=20), a different group of black South African healthy controls (n=10) and a group of black HIV-1-infected progressors (n=12).

The characteristics of cohort 2 are described in **Supplementary Table 2**. The HIV-1 controllers (controllers-2) included 6 individuals that met the criteria for elite controllers and included 5 of the 9 controllers from cohort 1 described above (TG4, TG11, Pru1, Pru2 and Pru3). HIV-1 infected progressors were recruited based on CD4+ T cell counts <250 cells/µl and VL >10,000 RNA copies/ml plasma, and were subsequently initiated on ART. The three groups [controllers-2, healthy controls (HCs-2) and progressors] did not differ significantly in age (*P*≥0.05 across all group comparisons), and although the progressors had markedly less females (58%) compared to the controllers-2 (85%) and HCs-2 (70%), the groups did not differ significantly (progressors vs. controllers-2, *P*=0.20; progressors vs. HCs-2, *P*=0.68; controllers-2 vs. HCs-2, *P*=0.63).

This study was approved by the University of the Witwatersrand Committee for Research on Human Subjects, and informed written consent was obtained from all of the participants.

### Plasma Viraemia Quantification and CD4+ T Cell Determination

HIV-1 RNA levels were quantified using one of two methods: (i) the COBAS^®^AmpliPrep/COBAS^®^TaqMan^®^ HIV-1 test v2.0 (Roche Diagnostic Systems, Indianapolis, IN, USA) with a lower detection limit of 20 HIV-1 RNA copies/ml or (ii) the Roche Amplicor RNA Monitor Assay (Roche) with a lower detection limit of 400 HIV-1 RNA copies/ml. CD4+ T cell counts were determined using the commercially available FACSCount System (Becton Dickinson, Franklin Lakes, NJ, USA).

### CCR5 Genotyping

The full-length *CCR5* gene sequence (∼9.2 kb) was determined for the 9 controllers (cohort 1) as described previously (31). A real-time assay was used for the detection of the *CCR2-V64I* polymorphism (17), thereby allowing genotyping of individuals according to the haplotypes described by Gonzalez et al. (32).

### CCR5 Quantification

EDTA-anticoagulated whole blood obtained from each of the study participants (cohort 1) was stained within one hour of blood collection. Four antibody panels were used for each sample to assess CCR5 expression on T, B and natural killer (NK) cells as well as granulocytes and monocytes. Furthermore, HLA-DR was included as a marker in a fifth panel to assess the extent of cell activation (i.e., percentage of HLA-DR-expressing cells) – this was carried out on all controllers and a subset (16/22) of the HCs. The detailed staining/flow cytometry method has been previously described (29). Briefly, the CCR5 antibody used was conjugated to phycoerythrin (PE) at a ratio of 1:1, thereby allowing for CCR5 quantification, as the mean number of CCR5 molecules per cell (CCR5 density), in addition to the percentage of CCR5-expressing cells within a cell subset. Quantification was carried out using the QuantiBRITE system (BD BioSciences) which is a set of four precalibrated beads to calibrate the fluorescence 2 (FL2) axis in terms of PE molecules.

### Soluble CD14 Quantitation

Plasma separated from EDTA-anticoagulated whole blood was diluted 1:1000 with phosphate buffered saline (PBS). The levels of sCD14 were quantified using the Human CD14 DuoSet ELISA Development System (R&D Systems), with a 62.5 pg/ml limit of detection, as per the manufacturer’s recommendations.

### Cytokine Production Measurement

Cytokine production assays were performed as previously described (33). Equal numbers of isolated peripheral blood mononuclear cells (PBMCs) were incubated for 20 h with or without phytohemagglutinin (PHA, 12.5 μg/ml). Concentrations within the harvested culture supernatants of the cytokines interleukin (IL)-2, IL-4, IL-6, IL-7, IL-8, IL-10, IL-12p70, granulocyte colony-stimulating factor (G-CSF), granulocyte-macrophage colony-stimulating factor (GM-CSF), interferon γ (IFN-γ) and tumour necrosis factor α (TNF-α) in addition to the CCR5 chemokine ligands Chemokine (C-C motif) ligand 3 (CCL3), CCL4 and CCL5 were determined either by ELISA (DuoSet ELISA Development Systems; R&D Systems, Minneapolis, Minnesota, USA) or Cytometric Bead Array (CBA) (BD BioSciences, San Jose, CA, USA). The samples were compared with protein standards.

CCL3, CCL4 and IL-8 were quantified in unstimulated and PHA-stimulated PBMCs by means of ELISA as described previously (33). The minimum detection levels were <10, 15.6 and 31.25 pg/ml for CCL3, CCL4 and IL-8, respectively. The remaining cytokine concentrations were determined by means of CBA. The CBA immunoassays were conducted as three separate multiplexes: (i) GM-CSF, G-CSF, IL-10 and IL-12p70; (ii) IL-7, IL-4 and IL-2 and (iii) TNF-α, IFN-γ and CCL5. The detection limits for the cytokines measured by means of CBA were as follows: TNF-α and CCL5, 1.25 pg/ml; IFNγ, 1.8 pg/ml; IL-4, IL-7, IL-12p70 and GM-CSF, 2.5 pg/ml; G-CSF and IL-10, 10 pg/ml; and IL-2, 11.2 pg/ml. All CBA immunoassay samples were analysed using a FACSCalibur (BD BioSciences) instrument and FCAP Array v1.0 software (SoftFlow, Hungary). Prior to analysis, the cytometer was calibrated using set-up beads according to the manufacturer’s instructions. All samples with concentrations below the minimum limit of detection (assay-specific for each cytokine) were assigned a value of zero, whereas those above the maximum detection limit, i.e. >2500 pg/ml, were repeated at an appropriate dilution. The cytokine production values were calculated as follows: cytokine production of PHA-stimulated PBMCs minus the cytokine production of unstimulated PBMCs, if within the assay detection levels, from the same individual.

### Absolute Counts of Cells in Blood Samples

TruCOUNT™ Tubes (BD Biosciences, San Jose, CA) were used to determine the absolute counts of lymphocytes and monocytes in blood. Assay conditions were as recommended by the manufacturer. Briefly, monoclonal antibodies were added with 50 μl whole blood to the lyophilized pellet containing a known number of fluorescent beads and the samples were prepared using a lyse/no wash procedure. Flow cytometric acquisition was performed on a FACSCalibur system (BD Biosciences). Data were analysed using FlowJo 7.6.1 (Tree Star, San Carlos, CA). Lymphocytes and monocytes were gated on a CD45 versus SSC dot plot, while beads were gated on a FL1 versus FL2 dot plot. Absolute counts (cells/μl) were calculated by the product of: [the number of events in the cell-containing region divided by the number of events in bead-containing region] and [number of beads per test (lot specific) divided by the test volume (50μl)].

T, B and NK cell counts were determined based on the proportions of total lymphocytes the respective cell subsets comprised, as determined from appropriate antibody panels used to quantitate CCR5 expression. Absolute counts of monocytes and lymphocyte subpopulations in the PBMC cultures (1.4 x 10^6^ cells total) were calculated based on the monocyte:lymphocyte ratio determined from the absolute counts.

### Relative CD4+ T Cell CCR5 mRNA Expression

CD4+ T cells were positively isolated from Ficoll-isolated PBMCs (cohort 2) using MACS^®^ cell separation technology with CD4+ Microbeads and MS columns (Miltenyi Biotec, Germany), according to manufacturer’s instructions. Cells were stored in 150 µl of *RNAlater* ^®^ solution (Life Technologies, California, USA) at -80°C until time of RNA extraction. Ice cold PBS (150 µl) was added to the *RNAlater* ^®^-cell suspension and the tube was then centrifuged for 6 minutes at 9000 g to pellet cells prior to extraction. RNA was extracted using the mirVana miRNA Isolation Kit (Ambion^®^, Life Technologies, California, USA), according to manufacturer’s instructions. Post extraction, RNA quality was assessed using the Agilent RNA 6000 Nano Kit and the Agilent 2100 Bioanalyzer system (Agilent Technologies, California, USA). All RNA samples had an RNA Integrity Number (RIN) greater than 7. The total RNA amount used in the cDNA synthesis was standardized to the sample with the lowest concentration. cDNA was synthesized using the Invitrogen Superscript III first strand synthesis system (Thermofisher Scientific, Massachusetts, USA), using both oligo-dT primers and random hexamers. A DNase digestion step was not included since our quantification assay probes all spanned exon-exon boundaries.

Synthesized cDNA was used as the template for gene-specific amplification using a predesigned gene expression hydrolysis probe assay for *CCR5* (Life Technologies: Hs00152917_m1). Two reference genes were used for normalization: ribosomal protein large, PO (*RPLPO*) (Life Technologies: Hs04189669_g1), and beta-actin (*ACTB*) (Life Technologies: Hs01060665_g1). Reactions (10 µl final volume) were performed in triplicate for each sample and were set up in 96-well plates, with each well containing 0.5 µl of the respective 20x Taqman Gene Expression Assay, 5 µl of 2x Taqman Gene Expression Mastermix (Life Technologies), 1 µl of cDNA and 3.5 µl of nuclease-free water (Ambion). Amplification was carried out on an Applied Biosystems 7500 Real-Time PCR system. The amplification settings included an initial holding stage at 95°C for 10 minutes and cycling stages (40 cycles) of 95°C for 15 seconds and 60°C for 40 seconds. A no template control (NTC) was included for each assay. Relative gene expression was calculated using the 2^∆Cq^ method, subtracting the average target gene C_q_ from the average reference gene C_q_ for each individual to get the ∆C_q_ value.

### Statistical Analysis

Fisher’s exact tests were performed using the Simple Interactive Statistical Analysis software (34) to test for differences in single nucleotide polymorphism (SNP) and haplotype frequencies found in the controllers and those found in a previously described cohort of healthy black South Africans (31) expanded by the recruitment of an additional six individuals (n=41). Two-sided tests were used and statistical significance was considered if *P*<0.05. Data presented as continuous variables, i.e., expression or production levels of the test molecules, were compared using a Mann-Whitney *U*-test. Spearman’s non-parametric correlations of select cell group CCR5 densities and a number of parameters (age, spontaneous PBMC CCR5 ligand production) were carried out. Mann-Whitney U-tests and Spearman’s correlations were conducted using GraphPad Prism v4.02 (GraphPad Prism Software, Inc, La Jolla, California, U.S.A.).

## Results

### CCR5 Gene Polymorphism Distribution

Assembled sequences of the *CCR5* gene including promoter, coding and 3’ untranslated region (UTR) regions, from 9 HIV-1 controllers were analysed for DNA polymorphisms, single nucleotide polymorphisms (SNPs) and indels. Across the entire 9.2 kb region sequenced, 36 SNPs and 4 indels were identified. No polymorphisms were found within the *CCR5* open reading frame (ORF). All polymorphisms identified had been previously reported in South African populations (31). The frequencies at which polymorphisms were found in controller individuals, along with the background population (an expanded HC cohort; n=41) frequencies are indicated in **Supplementary Table 3**. Four of the SNPs identified within the group of controllers (-4257A/C, -3886C/T, -1060C/T and +1823C/T) had previously been thought to be absent in black South African individuals (31). Among the identified polymorphisms, one SNP located in the 3′ UTR, rs3188094 (+2458A/C), was found at a significantly higher frequency within the controller group (27.8%) compared to the background population (8.5%) (*P*=0.038), with two controllers (Pru1 and Pru4; **Table 1**) being homozygous for the minor allele – homozygosity was not detected in the background population. Interestingly, this SNP does not form part of previously identified *CCR5* haplotypes (31, 32).

Individuals within the controller cohort were assigned to previously described haplogroups (32). No controllers were found to possess the *CCR5Δ32* (HHG*2) allele (**Table 1**). All haplotypes, with the exception of HHB, found in our background population were also found in the group of controllers. Haplotypes HHA, HHC, HHD, HHE, HHF*1, HHF*2 and HHG*1 were present at frequencies of 33% (6/18), 28% (5/18), 6% (1/18), 6% (1/18), 6% (1/18), 17% (3/18) and 6% (1/18), respectively. Haplotype frequencies did not differ between controllers and the background population.

### CCR5 Cell Surface Expression

#### HIV-1 Controllers Have Lower CCR5 Density on CD4+ T Cells and Monocytes Compared to HCs

**Figure 1:**
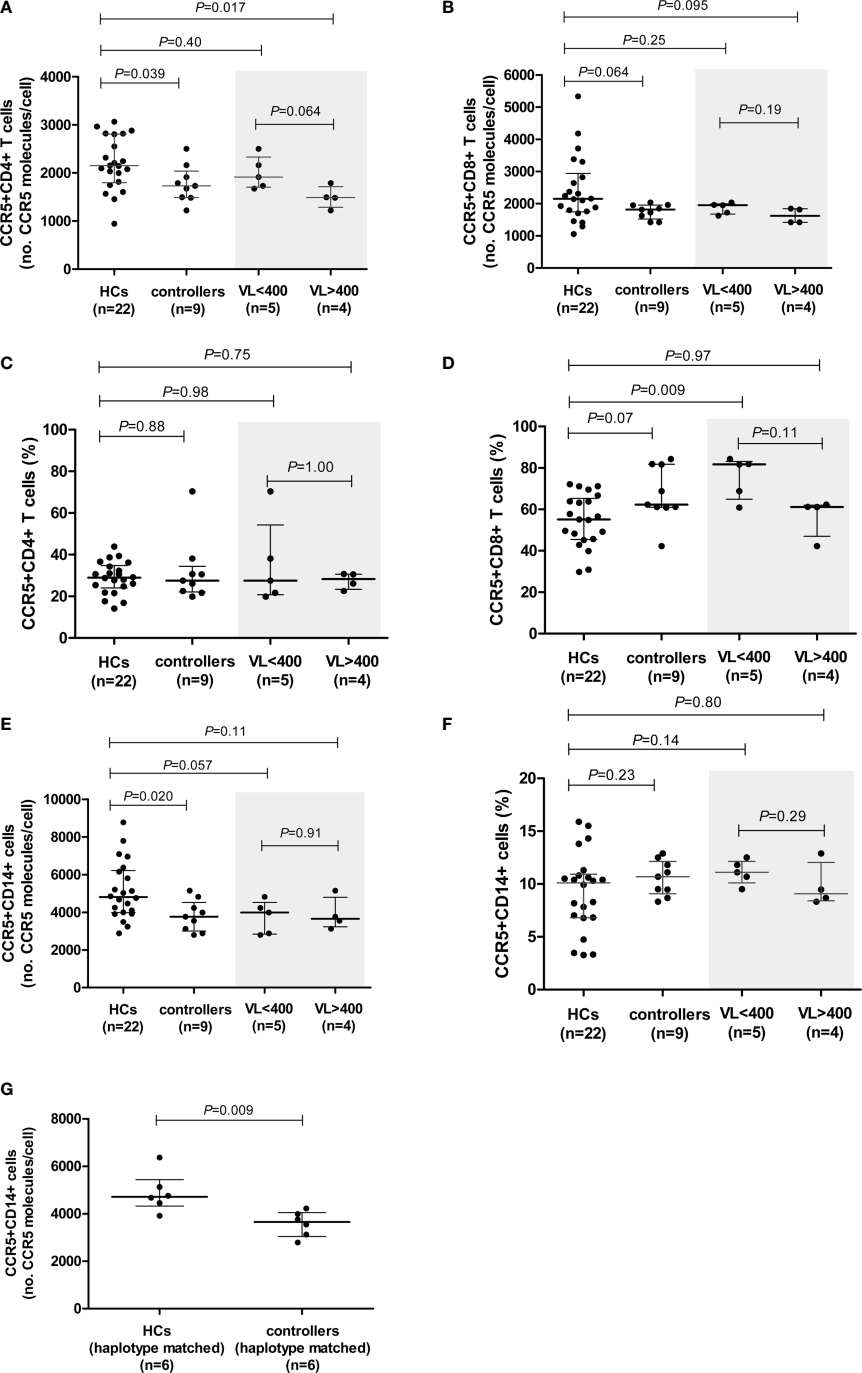
CCR5 expression (density and percentage CCR5-expressing cells) on CD4+ **(A, C)** and CD8+ **(B, D)** T cells and monocytes **(E, F)** in healthy controls (HCs) and HIV-1 controllers (cohort 1). CCR5 expression for controllers, stratified according to viral load (VL), VL<400 and VL>400 (RNA copies/ml), is shaded in grey. CCR5 density on monocytes in HCs and HIV-1 controllers matched for *CCR5* genotype **(G)**. Mann-Whitney *U*-tests were conducted to test for significance. The medians and interquartile ranges are shown by horizontal bars. *P* values and the number of individuals in each group are indicated.

Cell surface CCR5 expression of HIV-1 controllers was compared to that of HCs (29). CCR5 density was significantly lower on CD4+ T cells and on monocytes of controllers compared to HCs (*P*=0.039 and *P*=0.020, respectively, **Figures 1A, E**). Furthermore, there was a trend towards controllers expressing CCR5 at lower densities than HCs on CD8+ T cells (*P*=0.064, **Figure 1B**). It is interesting to note that the CCR5-density range on the CD8+ T cells of controllers was very narrow in comparison to HCs (1422-2035 versus 1055-5339 CCR5 molecules/cell, respectively). No differences in CCR5 density were noted in NK cell subsets between the two study groups (data not shown).

We next stratified our controller cohort into two groups according to the HIV VL: i) controllers with low VLs, i.e., <400 HIV-1 RNA copies/ml (n=5, range: <40 – 183 HIV-1 RNA copies/ml), and ii) controllers with higher VLs, i.e., >400 HIV-1 RNA copies/ml (n=4, range: 1155 – 6070 HIV-1 RNA copies/ml). Surprisingly, the CCR5 surface density was lower among controllers in the higher VL category. CCR5 density on CD4+ T cells in the VL<400 group was similar to that of the HCs, however, CCR5 density on CD4+ T cells of individuals with VL>400 was significantly lower than that of the HCs (*P*=0.017, **Figure 1A**). There was also a trend towards the VL>400 controllers expressing CCR5 at lower densities on CD4+ T cells compared to the VL<400 controllers (*P*=0.064, **Figure 1A**). The lower CD8+ T cell CCR5 density was mainly due to the VL>400 group of controllers (**Figure 1B**).

Controllers with VL<400 had similar CCR5 expression levels on monocytes to those observed in the VL>400 controllers (*P*=0.91, **Figure 1E**). No differences in CCR5 density on any cell subset relative to HCs were observed when controllers were stratified according to CD4+ T cell count < and >500 cells/μl (**Supplementary Figure 1**).

#### HIV-1 Controllers Have Higher Proportions of CCR5-Expressing CD8+ T Cells Relative to HCs

The proportion of CCR5-expressing CD4+ T cells did not differ between the controllers and HCs (*P*=0.88, **Figure 1C**). Similarly, the proportion of CCR5-expressing CD4+ T cells did not differ between the low and higher VL controller groups (*P*=1.00, **Figure 1C**). However, controllers had a strong trend of higher proportions of CCR5-expressing CD8+ T cells in comparison to HCs (*P*=0.07, **Figure 1D**). This relationship could be attributed to the VL<400 individuals, who had significantly higher frequencies of CCR5-expressing CD8+ T cells compared to that of the HCs (*P*=0.009, **Figure 1D**). Controllers with VL>400 however, had similar percentages of CCR5-expressing CD8+ T cells to the HCs (*P*=0.97, **Figure 1D**). Controllers with CD4+ T cell counts >500 cells/μl (CD4>500) had significantly higher proportions of CCR5-expressing CD8+ T cells compared to HCs (*P*=0.023, **Supplementary Figure 1**), however this was less significant than the VL<400 controller group comparison and is likely due to the three controllers with the highest frequency of CCR5-expressing CD8+ T cells (>80%) overlapping between the two groups - in fact the most significant difference in frequency of CCR5-expressing CD8+ T cells was seen when comparing the three controllers with VL<400 and CD4>500 to HCs (*P*=0.007, data not shown).

The percentage of CCR5-expressing monocytes did not differ between the controllers and HCs (*P*=0.23, **Figure 1F**), and did not differ either upon VL or CD4+ T cell count stratification (**Figure 1F** and **Supplementary Figure 1**, respectively).

#### Lower CCR5 Density on Monocytes of Controllers in Comparison to the HCs Remains Significant When Matched for CCR5 Promoter Haplotype and Age

We had previously reported a significant negative correlation between age and monocyte CCR5 density in the HCs (29). Since there was a strong trend of lower age in the HCs compared to the controllers (*P*=0.05), we selected a smaller, age-matched subgroup of the HCs (n=12) and again compared CCR5 density in monocytes, as well as CD4+ and CD8+ T cells (**Supplementary Figure 2**). Statistical significance was lost when monocyte CCR5 density between controllers and HCs (*P*=0.804), suggesting that age was likely a contributing factor to the significant lower density in controller monocytes in our original evaluation. Although the CD8+ T cell median for controllers was still lower than that of the HCs, the trend was lost (*P*=0.189). What is interesting however is that the CD4+ T cell significance was maintained and was slightly stronger (*P*=0.036).

To control for the possible influence of individual *CCR5* haplotypes and/or genotypes, we selected and compared a subgroup of individuals from the HC cohort that shared *CCR5* promoter genotypes with the controllers (only 6/9 controllers had genotypes that were present in the HC group). These subgroups consisted of the following genotypes (controllers:HCs): HHA/HHA (1:1); HHA/HHC (2:1); HHA/HHF*2 (2:1) and HHC/HHD (1:3). These subgroups did not differ with respect to age (*P*=0.748). The significant difference in CD4+ T cell CCR5 density was lost when CCR5 genotype-matched subgroups were compared; however, a weak trend was maintained (*P*=0.093, **Supplementary Table 4**). This could possibly be attributed to the small number of individuals in each group (n=6 in each). In contrast, the difference seen in monocyte CCR5 density between the controllers and HCs became more significant when age and genotype-matched subgroups were compared (*P*=0.009, **Figure 1G** and **Supplementary Table 4**). Given that these genotyped-matched groups did not differ in age, it is interesting that CCR5 density was so significantly reduced on controller monocytes relative to HCs, and suggests that although age is a determining factor for monocyte CCR5 density, there may be other mechanisms at play when one controls for age as well as CCR5 promoter strength (genotypes).

### HIV-1 Controllers Have Significantly Less CD4+ T Cell CCR5 mRNA Expression Compared to HCs and HIV-1 Progressors

**Figure 2:**
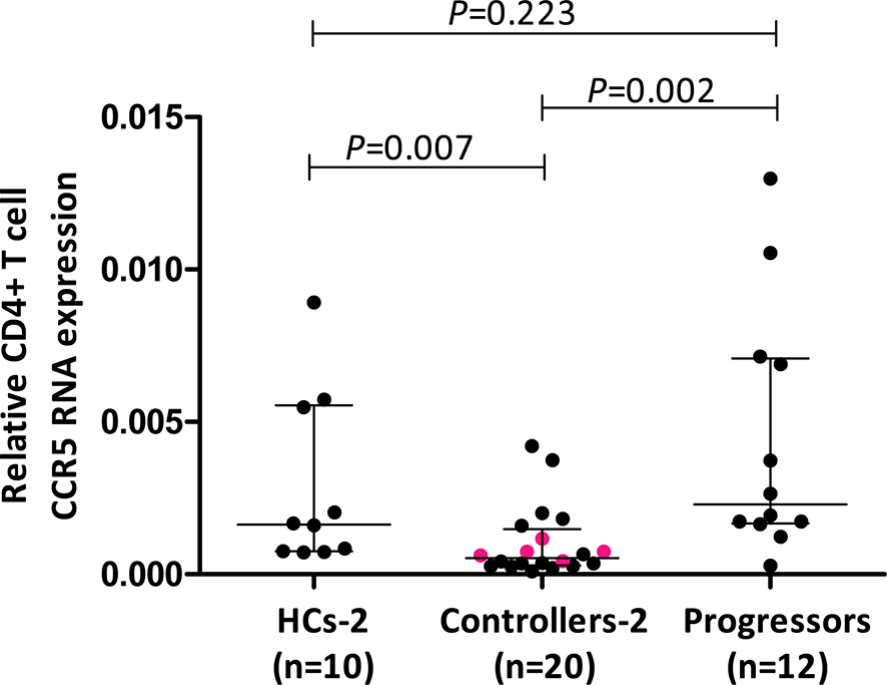
Comparison of relative CD4+ T cell CCR5 mRNA expression in healthy controls (HCs), HIV-1 controllers and HIV-1 progressors (cohort 2). Mann-Whitney *U*-tests were conducted to test for significance. The medians and interquartile ranges are shown by horizontal bars. *P* values and the number of individuals in each group are indicated. The pink coloured dots in the controller group indicate 5/9 controllers that were studied in cohort 1.

Results of relative CD4+ T cell CCR5 mRNA expression from a larger cohort of controllers (cohort 2) encompassing 5/9 controllers from cohort 1, compared to CD4+ T cell CCR5 mRNA from a different group of healthy controls (HCs-2, n=10) as well as HIV-1 progressors (n=12), are shown in **Figure 2**. Controllers had significantly less CCR5 mRNA expression compared to HCs (*P*=0.007) as well as to progressors (*P*=0.002). Interestingly, HCs and progressors did not differ significantly in terms of CD4+ T cell relative CCR5 mRNA expression (*P*=0.223). The 5 controllers that overlapped between cohort 1 and cohort 2 are shown in **Figure 2** as different coloured (pink) dots and were all situated below the medians for HCs and progressors.

### Cell Activation Was Higher In HIV-1 Controllers, Compared to the HCs in T Cell but Not NK and Monocyte Cell Subsets

**Figure 3:**
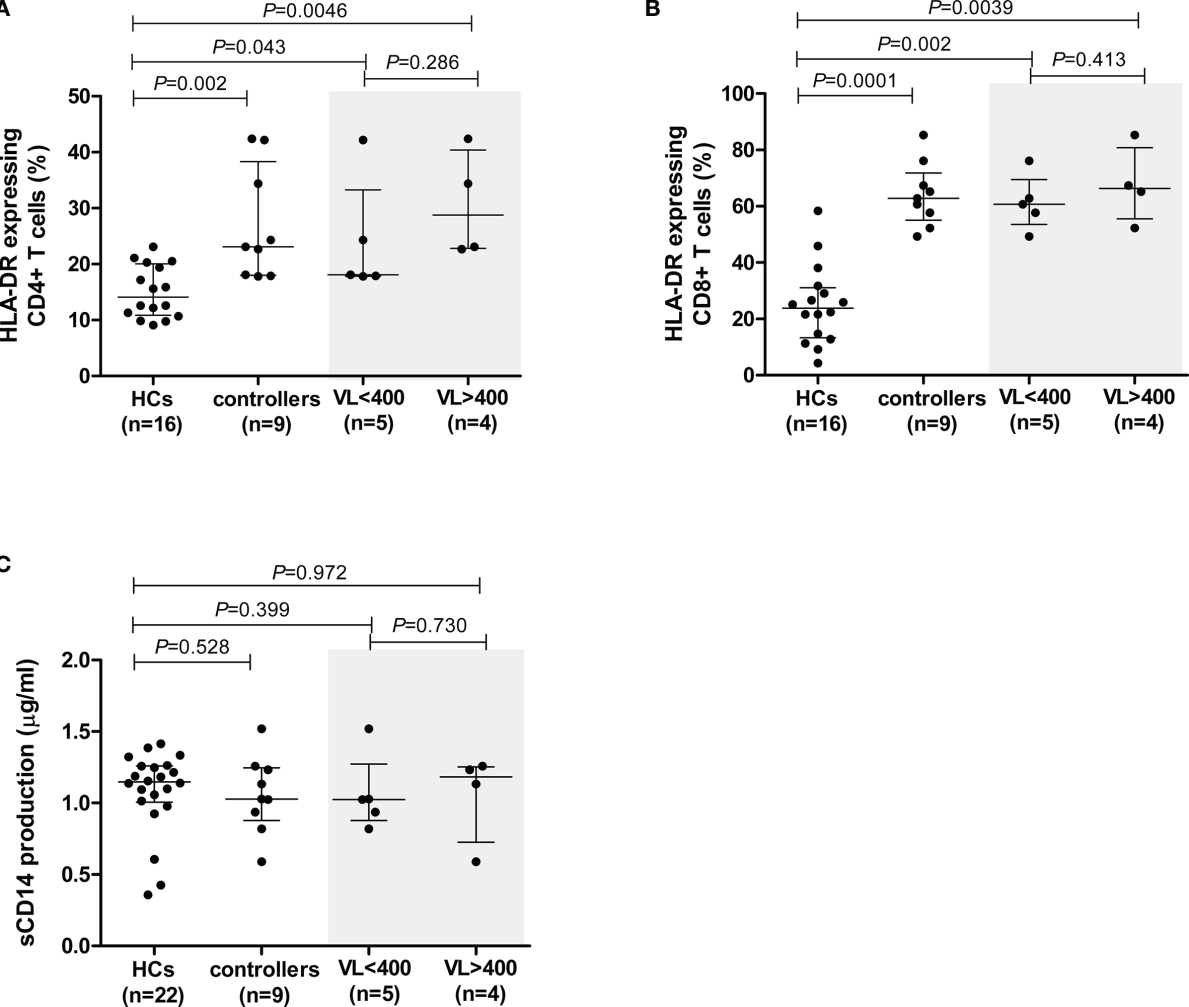
Cell activation levels in healthy controls (HCs) and HIV-1 controllers (cohort 1), as measured by the percentage of cells expressing HLA-DR for CD4+ T cells **(A)** and CD8+ T cells **(B)**, and sCD14 measured in plasma - a measure of monocyte cell activation **(C)**. Mann-Whitney *U*-tests were conducted to test for significance. The medians and interquartile ranges are shown by horizontal bars. *P* values and the number of individuals in each group are indicated. For the HLA-DR comparisons **(A, B)**, only 16 of the 22 HCs were included due to later incorporation of this marker in the study. Controllers stratified according to viral load (VL), VL<400 and VL>400 (RNA copies/ml) are shown within the grey shaded boxes.

Cell activation levels, as measured by the percentage of cells expressing HLA-DR, were significantly higher on CD4+ and CD8+ T cells in controllers compared to the HCs (*P*=0.002 and *P*=0.0001, respectively, **Figures 3A, B**). This is expected as a result of HIV-1 infection. However, no differences in cell activation were observed in NK cells (*P*=0.356, data not shown).

In controllers, cell activation levels were higher in CD8+ cells compared to CD4+ T cells. The median percentage of HLA-DR-expressing CD4+ T cells in controllers was 23.1%, whereas this value was considerably higher in CD8+ T cells, i.e., 62.8% (*P*<0.0001). When analysed according to VL-stratified groups, both controller groups demonstrated significantly higher CD4+ T cell cellular activation levels compared to HCs (*P*=0.043 for VL<400 and *P*=0.0046 for VL>400, **Figure 3A**). Similarly, both VL controller subgroups expressed HLA-DR on CD8+ T cell subsets at significantly higher levels than the HCs (*P*=0.002 for VL<400 and *P*=0.0039 for VL>400, **Figure 3B**). There was no difference in CD4+ or CD8+ T cell activation levels between the two controller VL subgroups (*P*=0.286 and *P*=0.413, respectively, **Figures 3A, B**). Similarly, stratification of controllers according to CD4+ T cell count showed both groups having significantly higher CD4+ T cell cellular activation levels compared to HCs (*P*=0.006 for CD4>500 and *P*=0.047 for CD4<500, data not shown) and both groups having significantly higher CD8+ T cell cellular activation levels compared to HCs (*P*=0.002 for CD4>500 and *P*=0.004 for CD4<500, data not shown). There was no difference in CD4+ or CD8+ T cell activation levels between the two controller CD4+ T cell subgroups (*P*=0.413 and *P*=0.730, respectively, data not shown).

Elevated plasma sCD14 in chronic HIV infection is associated with impaired immune restoration in response to ARV (35) as well as disease progression in both HIV-1 and HIV-2 infection (36–38). The sCD14 levels, measured in plasma samples from all study participants, were comparable between controllers and HCs (*P*=0.528, **Figure 3C**). Similarly, sCD14 production did not differ between controllers stratified according to VL (*P*=0.73, **Figure 3C**) or CD4+ T cell count (*P*=0.596, data not shown).

### PHA-Induced Production of the CCR5 Ligands Was Lower in HIV-1 Controllers Compared to the HCs

**Figure 4:**
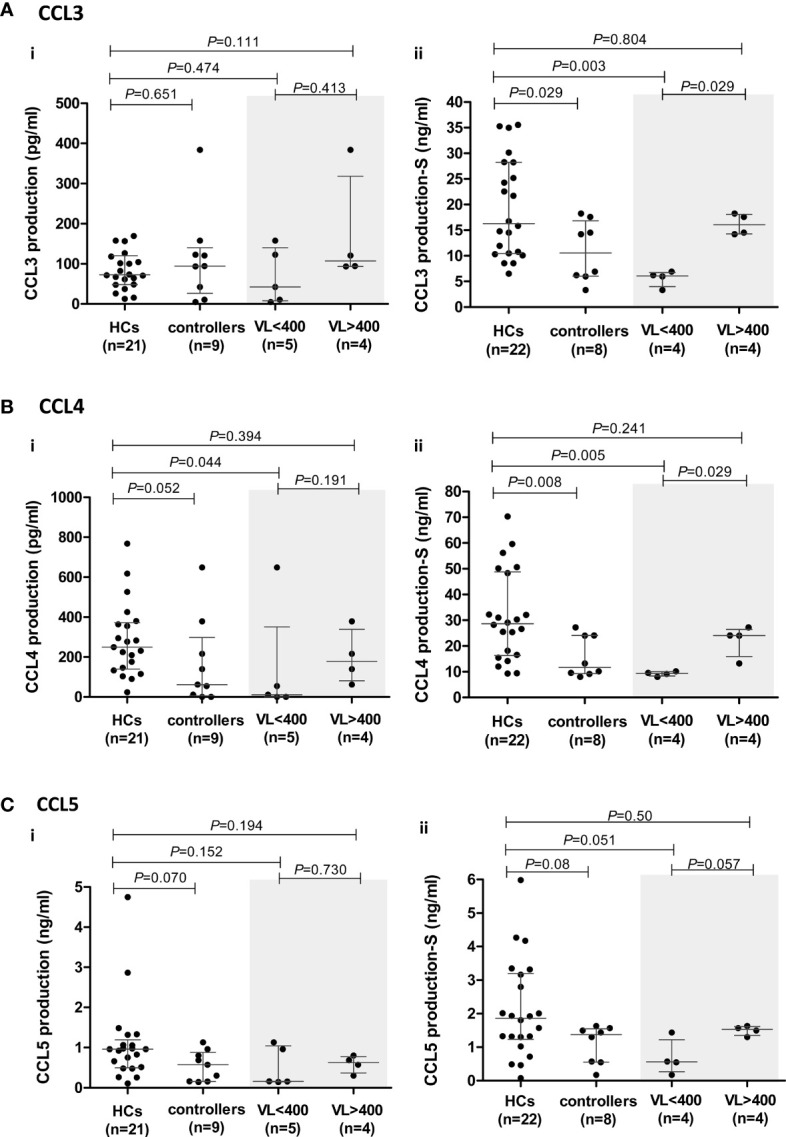
CCR5 ligand production by peripheral blood mononuclear cells (PBMCs) isolated from healthy controls (HCs) and HIV-1 controllers (cohort 1): CCL3 **(A)**, CCL4 **(B)** and CCL5 **(C)**. Overall cytokine production is shown for unstimulated (spontaneous) (i) and PHA-stimulated (S) (ii) PBMCs. Mann-Whitney *U*-tests were conducted to test for significance. The medians and interquartile ranges are shown by horizontal bars. *P* values and the number of individuals in each group are indicated. Due to insufficient sample, a single control individual was omitted from the unstimulated production assays. A Controller (TG4), considered an outlier, was omitted from the PHA-stimulated comparisons (hence, n=8). Chemokine production from controllers stratified according to viral load (VL), VL<400 and VL>400 (RNA copies/ml) are shown within the grey shaded boxes.

The chemokines CCL3, CCL4 and CCL5 were quantified in the supernatants of unstimulated and PHA-stimulated PBMCs following incubation at 37°C for 20 h. Spontaneous production of CCL3 and CCL4 was not different between controllers and HCs (**Figures 4Ai, Bi**), although there was a trend (*P*=0.052) towards lower CCL4 production in controllers, and stratification of controllers according to VL revealed that the VL<400 group had significantly lower production of CCL4 compared to the HCs (*P*=0.044, **Figure 4Bi**). Stratification according to CD4+ T cell count did not show any significant differences (**Supplementary Figure 3**).

Within the controller cohort, there was one individual (TG4) whose PHA-induced CCL3 and CCL4 production was well above that of the HCs and controller cohorts combined (CCL3: 44.95 ng/ml compared to 6.52 – 35.55 ng/ml in HCs and 3.33 - 18.24 ng/ml in the remaining controllers; CCL4: 82.77 nl/ml compared to 9.27 – 70.27 ng/ml in HCs and 8.06 – 27.24 ng/ml in the remaining controllers). We thus considered TG4 as an outlier and excluded him from the PHA-induced CCL3, CCL4 and CCL5 analyses (**Figures 4Aii**–**Cii**).

The PHA-induced production of CCL3 and CCL4 by controllers was significantly lower compared to the HCs (*P*=0.029 and P=0.008, respectively, **Figures 4Aii, Bii**). Stratification of controllers according to VL revealed that it was the VL<400 controllers that were driving the significant relationships seen, with these individuals having significantly lower CCL3 and CCL4 production compared to the HCs (*P*=0.003 and *P*=0.005, respectively, **Figures 4Aii, Bii**). When we stratified controllers based on CD4+ T cell count no significant differences were noted with CCL3, but the CD4>500 controllers had significantly lower CCL4 production compared to HCs (P=0.036, **Supplementary Figure 3**), however this was less significant than the VL<400 comparison.

Similarly, controllers produced lower levels of CCL5; however, this was not statistically significant (*P*=0.08, **Figure 4Cii**). This difference was more evident in unstimulated cultures where the median CCL5 production in the controllers (571.3 pg/ml) was lower than that of HCs (960.1 pg/ml, *P*=0.07, **Figure 4Ci**). PHA-stimulated PBMCs from VL<400 controllers also produced CCL5 at lower levels than HCs (*P*=0.051, **Figure 4Cii**) and PBMCs from controllers with VL>400 showed a strong trend of greater CCL5 production compared to the VL<400 group following PHA stimulation (*P*=0.057, **Figure 4Cii**). Stratification according to CD4+ T cell count did not show any significant differences (**Supplementary Figure 3**).

### PHA-Induced Production of Additional Cytokines

To investigate whether the lower chemokine production by PBMCs from controllers, compared to that by HCs, was restricted to the CCR5-ligand axis, we quantified the production of other cytokines: pro-inflammatory (IL-8, IFN-γ, TNF-α, G-CSF and GM-CSF), hematopoietic (IL-7, G-CSF and GM-CSF), T cell homeostatic (IL-2 and IL-4) and anti-inflammatory (IL-10) cytokines.

Spontaneous (unstimulated) PBMC production of only TNF-α and IL-8 were detectable in the assays used. Although controllers produced more TNF-α compared to HCs, this was not statistically significant (*P*=0.172, **Supplementary Figure 4A**). However, controllers with higher VLs (VL>400) produced significantly more TNF-α than the HCs (*P*=0.0095, **Supplementary Figure 4A**). A similar pattern was seen with IL-8, although not significant. The VL>400 subgroup produced more IL-8 compared to the HCs (*P*=0.129, **Supplementary Figure 4A**), and significantly higher than the VL<400 subgroup of controllers (*P*=0.029, **Supplementary Figure 4A**) – the TG4 outlier was excluded from the IL-8 analysis. These results suggest an association between VL and these two proinflammatory cytokines. No significant differences were seen when controllers were stratified according to CD4+ T cell count (data not shown).

**Figure 5:**
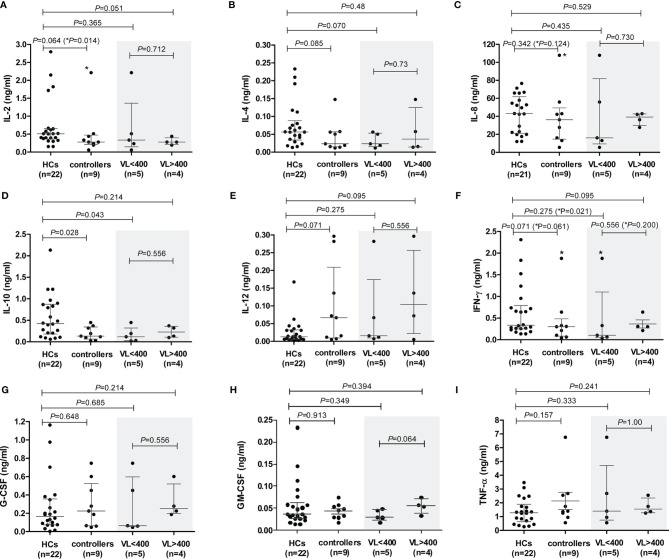
PHA-induced cytokine production by peripheral blood mononuclear cells (PBMCs) isolated from healthy controls (HCs) and HIV-1 controllers (cohort 1): IL-2 **(A)**, IL-4 **(B)**, IL-8 **(C)**, IL-10 **(D)**, IL-12 **(E)**, IFN-γ **(F)**, G-CSF **(G)**, GM-CSF **(H)** and TNF-α **(I)**. Mann-Whitney *U*-tests were conducted to test for significance. The medians and interquartile ranges are shown by horizontal bars. *P* values and the number of individuals in each group are indicated. Chemokine production from controllers stratified according to viral load (VL), VL<400 and VL>400 (RNA copies/ml) are shown within the grey shaded boxes. *P* values following the removal of the TG4 controller outlier as indicated with an asterisk (*) in **(A, C, F)**.

The levels of PHA-induced IL-7 were below detection levels for both cohorts. PHA-induced PBMC production of the cytokines IL-2, IL-4, IL-10 and IFN-γ was lower in controllers compared to the HCs (**Figures 5A, B, D, F**, respectively), attaining statistical significance only for IL-10 (*P*=0.028, **Figure 5D**). IL-2 showed a strong trend, which became strongly significant following the removal of the TG4 controller outlier (*P*=0.064 and *P*=0.014, respectively, **Figure 5A**). However, given that the amount of PHA-induced IL-2 produced by TG4 (2.2 ng/ml) fell within the range produced by the HCs (0.15 – 2.8 ng/ml), this result should be viewed with caution. Controllers with VL<400 showed a strong trend (*P*=0.070, **Figure 5B**) of lower PHA-induced IL-4 levels compared to the HCs (stronger than the total controller group comparison, *P*=0.085). In addition, a strong trend of lower PHA-induced IFN-γ production by controllers compared to HCs was observed (*P=*0.071, **Figure 5F**), which was also attributable to the VL<400 controllers; removal of the TG4 outlier resulted in a significantly lower production of IFN-γ by the VL<400 controllers relative to the HCs (*P=*0.021, **Figure 5F**) but, similar to IL-2, the PHA-induced IFN-γ produced by TG4 (1.88 ng/ml) fell within the range produced by the HCs (0.14 -2.31 ng/ml) and thus should also be viewed with caution. IL-12 was the only cytokine that showed a trend of higher PHA-induced production in controllers relative to the HCs (*P=*0.071, **Figure 5E**). No differences in PHA-induced production of IL-8, G-CSF, GM-CSF and TNF-α were noted between the two study groups (**Figures 5C, G, H, I**, respectively). Stratification of controllers according to CD4+ T cell count did not show any significant or stronger trends than stratification according to VL, with the exception of IL-10 where controllers with CD4<500 showed a strong trend of lower IL-10 compared to HCs (*P*=0.06, **Supplementary Figure 5**) and IL-12, where controllers with CD4>500 showed a strong trend of higher IL-12 production compared to HCs (*P*=0.057, **Supplementary Figure 5**).

### HIV-1 Controller PBMCs Had Higher Proportions of T Cells and Lower Proportions of NK Cells Compared to HC PBMCs

**Figure 6:**
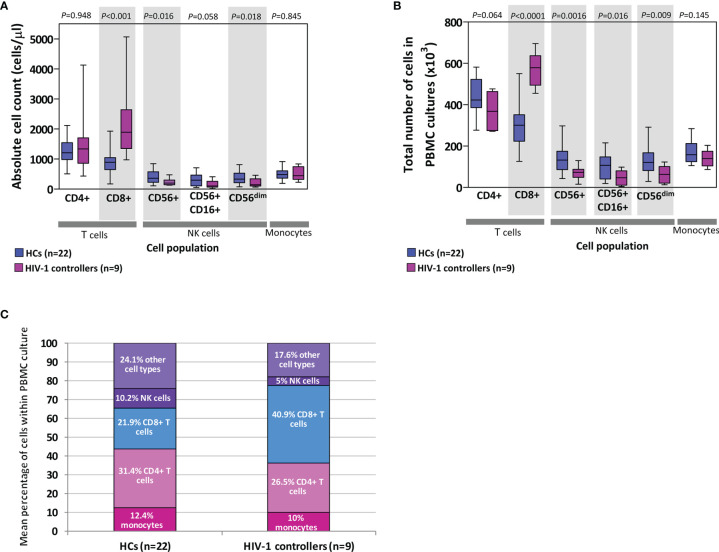
Absolute counts of cells (lymphocytes and monocytes) in blood **(A)** as well as extrapolated number of cells used in peripheral blood mononuclear cell (PBMC) cultures **(B)** in healthy controls (HCs) and HIV-1 controllers (cohort 1). Shaded grey boxes indicate significant differences between the two groups with *P* values as indicated (Mann-Whitney *U* tests). Box-whisker plots depict the median (horizontal black line), 25^th^ and 75^th^ percentiles (margins of the box) and the 10^th^ and 90^th^ percentiles (whiskers). Proportional representation of different cell types within PBMCs isolated from HCs and controllers determined using the mean values from the respective groups **(C)**. Cells referred to as “other cell types” include B cells, double-negative T cells, basophils and dendritic cells.

PBMCs are comprised of monocytes and lymphocytes, including T (CD4+ and CD8+), B and NK cells. We calculated the expected number of each of these cell types within the PBMCs used in chemokine/cytokine production assays by extrapolating from absolute cell counts determined from whole blood. **Figure 6** shows comparisons of both the absolute counts (**Figure 6A**), the calculated total number of cells used in the PBMC cultures (**Figure 6B**), and a comparison of the mean percentage of different cell types within the PBMC cultures (**Figure 6C**).

Controllers had similar absolute CD4+ T cell counts to HCs (P=0.948, **Figure 6A**), and although not statistically significant, controllers had fewer CD4+ T cells compared to the HCs in the total number of cells used in the PBMC cultures (*P*=0.064, **Figure 6B**). This CD4+ T cell deficit was offset by the significantly higher number of CD8+ T cells compared to the HCs, in both the absolute count and PBMC culture comparisons (*P*<0.001 and P<0.0001, **Figures 6A, B**, respectively). CD8+ T cell expansion is expected in individuals infected with HIV-1. Controllers were also found to have lower numbers of NK cells (CD56+, CD16+CD56+ and CD56^dim^) than HCs, evident as both absolute counts and NK cell numbers in PBMC cultures (*P*=0.016, *P*=0.058, *P*=0.018 and *P*=0.0016, *P*=0.016, *P*=0.009, **Figures 6A, B**, respectively). No differences were noted between the two groups in either absolute monocyte counts in blood or in PBMC cultures (*P*=0.845 and *P*=0.145, **Figures 6A, B**, respectively). **Figure 6C** indicates the proportional representation of different cell types within PBMCs from controllers and HCs – differences seen in the production of specific cytokines may be in part attributed to differences in representation of particular cell types.

## Discussion

The vast variation in the rates of HIV-1 disease progression among individuals can be attributed to viral, genetic and immunological factors. A number of host genetic factors associated with delayed disease progression have been identified [Reviewed in (39, 40)]. Among the genetic factors known to associate with delayed disease progression are genetic polymorphisms of *CCR5*, the principal HIV-1 coreceptor, and gene copy number variation of its ligands, CCL3L and CCL4L (41–43). In this study, we compared features important in the CCR5 coreceptor-ligand axis between two groups of black South African individuals—HIV-1 controllers who are able to control HIV-1 infection for extended periods of time in the absence of antiretroviral treatment and healthy controls. We included measures of CCR5 cell surface density, immune activation (proportions of CD4+ and CD8+ T cells expressing CCR5 and HLA-DR, and plasma levels of sCD14) and the capacity of mononuclear cells to produce chemokine/cytokines in response to PHA. Despite the small number of controllers used in these comparisons, these individuals have been closely monitored over many years and 8/9 controllers have been able to control disease in the absence of ART for ≥11 years, with two of these individuals for at least 20 years. In addition a larger cohort of HIV-1 controllers (including 5 of the 9 controllers studied in detail) was assessed for CCR5 mRNA expression in CD4+ T cells and compared to both healthy controls and HIV-1 progressors.

Although 36 SNPs and four indels were identified within the controllers (cohort 1), these were restricted to the noncoding regions of the *CCR5* gene and all had previously been identified. In a recent study, we investigated various *CCR5* regulatory genetic variants in a larger cohort of HIV-1 controllers (which included 8 of the 9 controllers from this study) and HIV-1 progressors (44). While select promoter haplotypes and variants were significantly over-represented in HIV-1 progressors relative to the controllers in that study, these same variants did not differ between this smaller cohort of controllers and healthy controls in the current study (HCs were not included in the Koor et al. (44) study). A 3’ UTR SNP (rs3188094; +2458A>C) was significantly more prevalent in the controllers compared to the HC cohort in this study (27.8% versus 8.5%), with two controllers being homozygous for the minor allele. However, representation of this SNP in the larger controller cohort (9.86%) is more aligned with the HC representation (44). It is interesting to note however that this +2458A>C SNP is a relatively rare SNP and data from the 1000 Genomes Project (45) shows that it is a predominantly African-based variant with 4% representation in the total African population group. Furthermore homozygosity for the minor allele is extremely rare (0.002% in the total African population – only one individual out of 661 individuals) – thus the black South African population exhibits a relatively high representation of this variant. Although not significant, representation of the +2458A>C SNP was also found to be lower in black South African HIV-1 infected progressors (4.7%) compared to controllers (44) and may be worth investigating further in terms of its role in CCR5 expression or function – preliminary analysis using a miRNA target prediction tool (http://www.targetscan.org/) revealed the major allele of the +2458A>C SNP to sit within the predicted binding site of the hsa-miR-376b-3p miRNA in the CCR5 3’UTR (data not shown).

HIV-1 infection is associated with increased CCR5 density on T cells, particularly CD4+ T cells (46, 47); however, we observed the opposite in that HIV-1-infected controllers expressed CCR5 at lower densities compared to HCs within these same cell subsets. Furthermore, controllers also exhibited significantly lower relative abundance of CD4+ T cell CCR5 mRNA compared to both HCs and HIV-1 progressors. The beneficial effects of reduced CCR5 expression on CD4+ T cells has been highlighted in simian immunodeficiency virus (SIV) infection models, where natural SIV hosts that do not develop AIDS-like symptoms express CCR5 on remarkably lower proportions of CD4+ T cells in blood, lymph nodes and mucosal tissues compared to humans and non-natural SIV hosts such as rhesus macaques (48).

The role of CCR5 expression in the natural control of HIV-1 has not been extensively studied. However, there have been some reports, conducted in predominantly European/Caucasian populations, which support our findings in a sub-Saharan black population. A study conducted on 9 slow progressors reported significantly lower CCR5 densities on CD4+ T cells of slow progressors compared to uninfected controls and HIV-1-infected normal progressors as well as rapid progressors (13). Furthermore, and in agreement with our study, no difference was seen between slow progressors and HCs in terms of the percentage of CCR5-expressing CD4+ T cells (13). Another study conducted on 12 HIV-1 controllers also found lower CCR5 density on CD4+ central memory T cells and a lower percentage of central memory, but not effector memory, CCR5-expressing CD4+ T cells in HIV-1 controllers relative to uninfected donors (49). Interestingly, the lower CCR5 density seen on the CD4+ T cells of our controllers relative to HCs was largely attributed to those with the higher VLs (>400 RNA copies/ml). This suggests that CCR5 density may be a greater contributor to HIV-1 control in the context of higher viraemia, at least in these particular individuals, as seen in the SIV model of infection.

In a more recent and larger study by Gonzalo-Gil et al. (50), a subgroup (n=21) of elite and viraemic controllers, who were identified with *in vitro* CD4+ T cell resistance to R5-tropic HIV-1 (ECs_r_/VCs_r_), had lower CD4+ T cell CCR5 densities and lower proportions of CCR5-expressing CD4+ T cells following CD4+ T cell stimulation relative to HCs and ECs/VCs who did not express the resistance phenotype (50). In addition, these ECs_r_/VCs_r_ has significantly decreased CCR5 mRNA expression levels in activated CD4+ T cells relative to HCs and ECs/VCs who did not express the resistance phenotype. Although this study differs from the present study in a number of parameters, it serves to corroborate the vital role of lower CD4+ T cell CCR5 expression in HIV-1 control.

Cell surface expression of CCR5 on monocytes plays an important role in HIV-1 infection. CCR5 expression correlates directly with the differentiation of monocytes to macrophages (51, 52). Although controllers had significantly lower monocyte CCR5 density compared to HCs, when we age matched a smaller group of HCs and compared CCR5 density between the two groups, the relationship was lost, however the significant relationship for CD4+ T cells was maintained. Given that we have previously shown a significant negative correlation between age and monocyte density in the HC cohort (29), the lower CCR5 density on monocytes is likely influenced by age and needs to be investigated in larger age-matched cohorts. However, we have also previously demonstrated *CCR5* haplotype-associated differences in CCR5 expression within healthy black South African individuals (17). When we matched controllers and HCs for *CCR5* genotypes (and age), CCR5 density on controller monocytes was distinctly significantly less (*P*=0.009) compared to HCs - given the strong influence of age on monocyte CCR5 density seen in the larger group comparison, it is difficult to interpret this highly significant relationship, and it is possible that it represents a chance result of smaller groups being compared. Nevertheless, this may be an important finding that should be interrogated in larger, haplotype and age matched cohorts. Very little, to our knowledge, has been reported about the role of monocyte CCR5 expression levels in natural HIV-1 control. In the Gonzalo-Gil et al. (50) study, CCR5 expression in macrophages derived from monocytes (MDMS) – measured as the levels of CCR5-specific RNA and percentage of CCR5-expressing CD14+ cells - was not different from healthy controls in the group of ECs/VCs with CD4+ T cells that displayed resistance to R5 tropic HIV-1. However, this result is not strictly comparable to our study and furthermore, they did not report on CCR5 density in MDMS.

Previous studies suggest that CCR5 receptor expression remains stable in HIV-1-uninfected individuals over multiple time points despite the wide range of variability that exists between individuals (12, 46). Furthermore, in the context of HIV-1 infection, CCR5 cell surface density levels correlate positively with the levels of immune activation (proportions of CD38^hi^-expressing CD8+ T cells), yet initiation or interruption of ART affects levels of immune activation but not CCR5 density (27). These data suggest a constitutive or inherent level of CCR5 cell surface expression within individuals, which is unaffected by the individual’s state of immune activation. Overall, high CCR5 density more likely predisposes to the likelihood of greater immune activation (expansion of proportions of HLA-DR, CD38 or CCR5-expressing CD4 or CD8 T cells) and disease progression, rather than the reverse i.e. the cause and consequence argument. As expected, individuals infected with HIV-1 (controllers) demonstrated higher T cell activation levels, as measured by the percentage of HLA-DR-expressing cells, compared to the HCs. Given that increased cell activation is associated with increased CCR5 expression (46, 53–55), it is intriguing that these same individuals expressed CCR5 at densities lower than that of HCs on the same cells. CD38 is widely used as a marker of T and B cell activation. In a cross-sectional study of individuals with normal progressing HIV-1 infection, we previously demonstrated significantly higher percentage of CD38+CCR5+ lymphocytes compared to healthy controls (56). On re-examination of these same data, the percentage of CCR5-expressing CD8+ T cells from HIV-1-infected individuals was found to correlate positively with the percentage of CD38-expressing CD8+ T cells (*P*=0.005, r=0.54; S. Shalekoff, unpublished data). Therefore, the percentage of CCR5-expressing CD8+ T cells could also be used as a surrogate marker for the extent of cell activation. The expansion of this same cell subset, but not of CCR5-expressing CD4+ T cells, was substantially higher in controllers compared to the HCs, which reflects the persistently higher cellular activation observed in HIV-1 controllers. However, we observed lower CD4+ T cell CCR5 density and mRNA expression in controllers compared to HCs. These findings suggest that, rather than controllers having the ability to downregulate CCR5 expression despite high levels of activation, controllers are comprised of individuals who are inherently low CCR5-expressors. As such, their immune cells are more quiescent and not as “activatable” as other individuals who progress more rapidly. Additional support for this theory is provided by the Gonzalo-Gil et al. (50) study. The CD4+ T cell R5 resistance phenotype of ECs_r_/VCsr, associated with the downregulation of ≈500 kb region of Chromosome 3p21 encompassing *CCR5* and *CCR2* (among other genes) was also observed in family members of an index VC. These family members also displayed *CCR5* and *CCR2* downregulation - thereby suggesting an inherited genetic determinant of lower CCR5 expression (50). CCL3, CCL4 and CCL5 are the natural ligands for the CCR5 receptor and are known to inhibit replication of CCR5-restricted HIV-1 variants (57, 58). While the anti-HIV-1 activity of the CCR5 ligands is mainly attributed to competitive binding to CCR5 (57), a role for these β-chemokines in inhibition of post-entry steps of the HIV-1 life cycle has also been reported (59). Although several studies have investigated the influence of HIV-1 infection and disease progression on circulating levels of CCL3, CCL4 and CCL5, results have been contradictory. There is a lack of consensus on whether individuals infected with HIV-1 produce these chemokines at higher, lower or equivalent levels compared to uninfected individuals, as measured in plasma or serum samples (60–62). Furthermore, there is a lack of consensus on whether or not cellular production and/or circulating levels of the CCR5 ligands correlate with disease progression (60, 62–67). These results are difficult to interpret due to a lack of homogeneity in patient selection as well as differences in study design. In addition, assays for their quantitation do not distinguish between the different chemokines and their isoforms (produced from different genes, present in variable copy numbers and subject to post-translation modifications that alter function/receptor binding), which could mask the true relationships of isoforms that may matter. In the context of mother-to-child HIV-1 transmission, we have shown that elevated levels of mitogen-induced CCL3 production (and to a less extent CCL4) by infant cord-blood mononuclear cells was associated with protection from intrapartum infection (68), suggesting that the levels of these ligands may play different roles depending on HIV acquisition versus disease progression.

If activation levels correlate with β-chemokine production, one might expect spontaneous PBMC production of CCL3, CCL4 and CCL5 to be higher for controllers than HCs; however, CCL3 production was comparable between the two groups, and CCL4 and CCL5 production of the controllers was lower than the HCs. Stimulation with PHA resulted in lower PBMC production of all three CCR5 ligands in controllers compared to that of the HCs, but this was only significantly lower for CCL3 and CCL4. In agreement with our results, activated CD4+ T cells from ECs/VCs that displayed the R5 HIV-1 resistance phenotype discussed earlier (ECs_r_/VCs_r_) also produced significantly less CCL3 and CCL4 compared to healthy controls (50). Interestingly, PBMCs from controllers with low VLs (VL<400) produced CCL3, CCL4 and CCL5 at lower levels than those from individuals with higher VLs and although not statistically significant, there seemed to be a similar trend in unstimulated cells. The former group also demonstrated slightly lower activation levels than the higher VL group, as measured by the percentage of cells expressing HLA-DR on CD4+ and CD8+ T cells. It could be argued that higher plasma concentrations of the CCR5 ligands could be responsible for increased internalization of the CCR5 receptor and hence lower CCR5 density, and controllers with higher VLs would by virtue of higher activation have higher plasma ligand concentrations and thus lower CCR5 density. However, CCR5 density on CD4+ T cells, CD8+ T cells and monocytes did not significantly correlate with spontaneous CCL3, CCL4 or CCL5 production in HCs or controllers (data not shown). Furthermore the lower CD4+ T cell CCR5 mRNA expression seen in controllers relative to both HCs and progressors points to a transcriptional downregulation rather than receptor internalization as the predominant cause of lower CCR5 density.

CD26/DPP4-mediated proteolysis of CCL3L1 and CCL5 results in a strong affinity of these isoforms for binding to CCR5, which also display potent anti-HIV-1 activity *in vitro* [reviewed in (69)]. In a recent study we conducted, HIV-1 controllers had similar levels of specific CD26/DPP4 activity and percentages of CD26/DPP4+ T cells compared to HCs, but significantly higher levels than HIV-1 progressors (70). Taken together, we postulate that the relative abundance of more effective anti-HIV chemokine isoforms is greater in controllers compared to progressors, due to ineffective CD26/DPP4 proteolysis in the latter, which accordingly is also associated with increased inflammation.

IL-10 was the only cytokine that was produced at significantly lower levels by PHA-stimulated PBMCs from controllers relative to HCs, without removal of outliers, and in fact remained significant post removal of a high-producing IL-10 HC outlier. Numerous reports have highlighted the importance of circulating IL-10 levels in the course of HIV-1 infection. Although high IL-10 levels, associated with *IL-10* single nucleotide polymorphisms, provide protection against acquiring HIV-1, as demonstrated in a study of high-risk South African women who were HIV-1-uninfected when enrolled into the study (71), lower IL-10 levels appear to provide beneficial effects in the chronic phase of HIV-1 infection. Blockade of the IL-10 pathway is capable of restoring HIV-1-specific T cell responses (72, 73). In addition, individuals infected with HIV-1 with *IL-10* genotypes associated with lower IL-10 production, demonstrate a trend towards attenuated CD4+ T cell loss (74). However, the effects of IL-10 on HIV-1 pathogenesis seem to differ according to the stage of infection (71, 72, 74), thus indicating a complex relationship between IL-10 levels and HIV-1 disease progression. Although PHA-induced production of IL-10 by PBMCs has been reported as increased in individuals infected with HIV-1 relative to HCs (75), comparable IL-10 production between HIV-1 long-term non-progressors and uninfected individuals has been reported, whereas individuals with progressive infection maintained significantly higher IL-10 production (76). Furthermore, IL-10 production correlates positively with disease progression (73, 77). Together, this suggests that the clinical progression status of an individual infected with HIV-1 is likely to result in differences in antigen-induced production of IL-10. Furthermore, monocytes are major producers of IL-10 in both individuals with and without HIV-1 infection [Reviewed in (78)]. Plasma levels of sCD14, indicative of the extent of *in vivo* monocyte activation, did not differ between our controllers and HCs. In addition, monocyte numbers in *in vitro* PHA-stimulated cultures did not differ, suggesting that controllers produce less IL-10 independently of monocyte numbers or the level of their activation.

This study has a number of limitations. We only included a small number of controllers, who were also heterogeneous based on definitions of HIV-1 control, in the cell-based experiments. Using larger cohorts that are better matched for age, and including ART-treated HIV-1-infected progressors in future studies will shed further light on the current findings. We also used HLA-DR alone as a marker of activation - the use of CD38 in addition to HLA-DR would have been more informative, and more detailed analysis of different subsets of activated cells in the context of CCR5 density warrant further investigation. The comparison of CCR5 density directly between activated and non-activated CD4+ and CD8+ T cells would have provided more compelling evidence for the postulated inherent predisposition to lower CCR5 density. We were unable to do this based on the HLA-DR marker being in a separate 4-colour flow cytometry panel.

In summary, in this study we demonstrate reduced CCR5 cell surface density on CD4+ T cells and reduced induced cellular levels of the CCR5 ligands (CCL3, CCL4 and CCL5) and IL-10, in a small group of black HIV-1 controllers compared to HCs. In addition, we show lower CCR5 mRNA expression in CD4+ T cells in a larger group of controllers relative to both HCs and HIV-1-infected progressors. Importantly, this pattern of lower CCR5 expression in CD4+ T cells has also been shown for Caucasian HIV-1 controllers, is independent of the presence of the *CCR5Δ32* deletion (13, 50), and is likely to involve a transcriptional downregulation of a large region of the chromosome encompassing *CCR5*, which appears to be genetically predetermined (50). A genetically predetermined lower CCR5 expression is in keeping with our findings and thus probably involves a mechanism that is shared among ethnically divergent population groups – this is an important finding and supports continued investigation into the underlying mechanism responsible for this phenomenon, which could inform future HIV cure efforts, particularly in sub-Saharan Africa, where cure interventions are most needed.

## Data Availability Statement

The original contributions presented in the study are included in the article/**Supplementary Material**. Further inquiries can be directed to the corresponding author.

## Ethics Statement

This study was approved by the University of the Witwatersrand Committee for Research on Human Subjects. The patients/participants provided their written informed consent to participate in this study.

## Author Contributions

AP: Conducting experiments, analysis and writing. MP: Analysis and writing. GWK: Conducting experiments. AB: Conducting experiments. SS: Conducting experiments, review and editing. RL: Conducting experiments, review and editing. PI: Resources (cohort). CTT: Conceptualization, funding acquisition, writing. All authors contributed to the article and approved the submitted version.

## Funding

This work is based on the research supported by grants awards from South African Research Chairs Initiative of the Department of Science and Technology and National Research Foundation of South Africa (grant no. 84177), the South African Medical Research Council (Self-Initiated Research Grant), the Strategic Health Innovation Partnerships (SHIP) Unit of the South African Medical Research Council (a grantee of the Bill & Melinda Gates Foundation) and the Poliomyelitis Research Foundation (PRF).

## Conflict of Interest

The authors declare that the research was conducted in the absence of any commercial or financial relationships that could be construed as a potential conflict of interest.

## Publisher’s Note

All claims expressed in this article are solely those of the authors and do not necessarily represent those of their affiliated organizations, or those of the publisher, the editors and the reviewers. Any product that may be evaluated in this article, or claim that may be made by its manufacturer, is not guaranteed or endorsed by the publisher.
