# Supplementary material for: Reduced CCR5 Expression and Immune Quiescence in Black South African HIV-1 Controllers
Source: Front Immunol. 2021 Dec 20;12:781263. doi: 10.3389/fimmu.2021.781263 (PMC8720782; doi:10.3389/fimmu.2021.781263)
Supplement: Supplementary file 1 [file Presentation_1.pptx]

## Slide 1
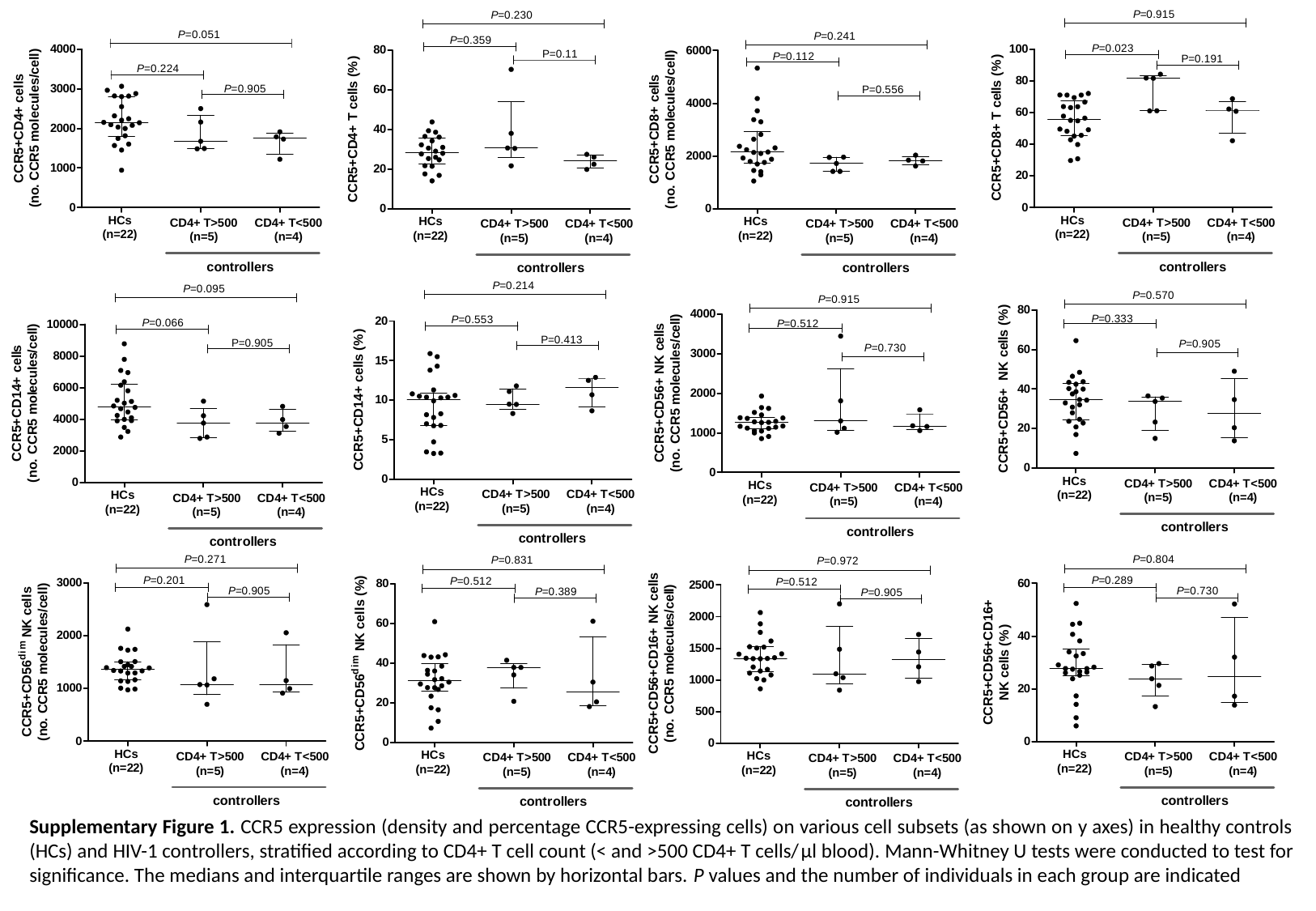

Supplementary Figure 1. CCR5 expression (density and percentage CCR5-expressing cells) on various cell subsets (as shown on y axes) in healthy controls (HCs) and HIV-1 controllers, stratified according to CD4+ T cell count (< and >500 CD4+ T cells/μl blood). Mann-Whitney U tests were conducted to test for significance. The medians and interquartile ranges are shown by horizontal bars. P values and the number of individuals in each group are indicated
