# Supplementary material for: Reduced CCR5 Expression and Immune Quiescence in Black South African HIV-1 Controllers
Source: Front Immunol. 2021 Dec 20;12:781263. doi: 10.3389/fimmu.2021.781263 (PMC8720782; doi:10.3389/fimmu.2021.781263)
Supplement: Supplementary file 2 [file Presentation_2.pptx]

## Slide 1
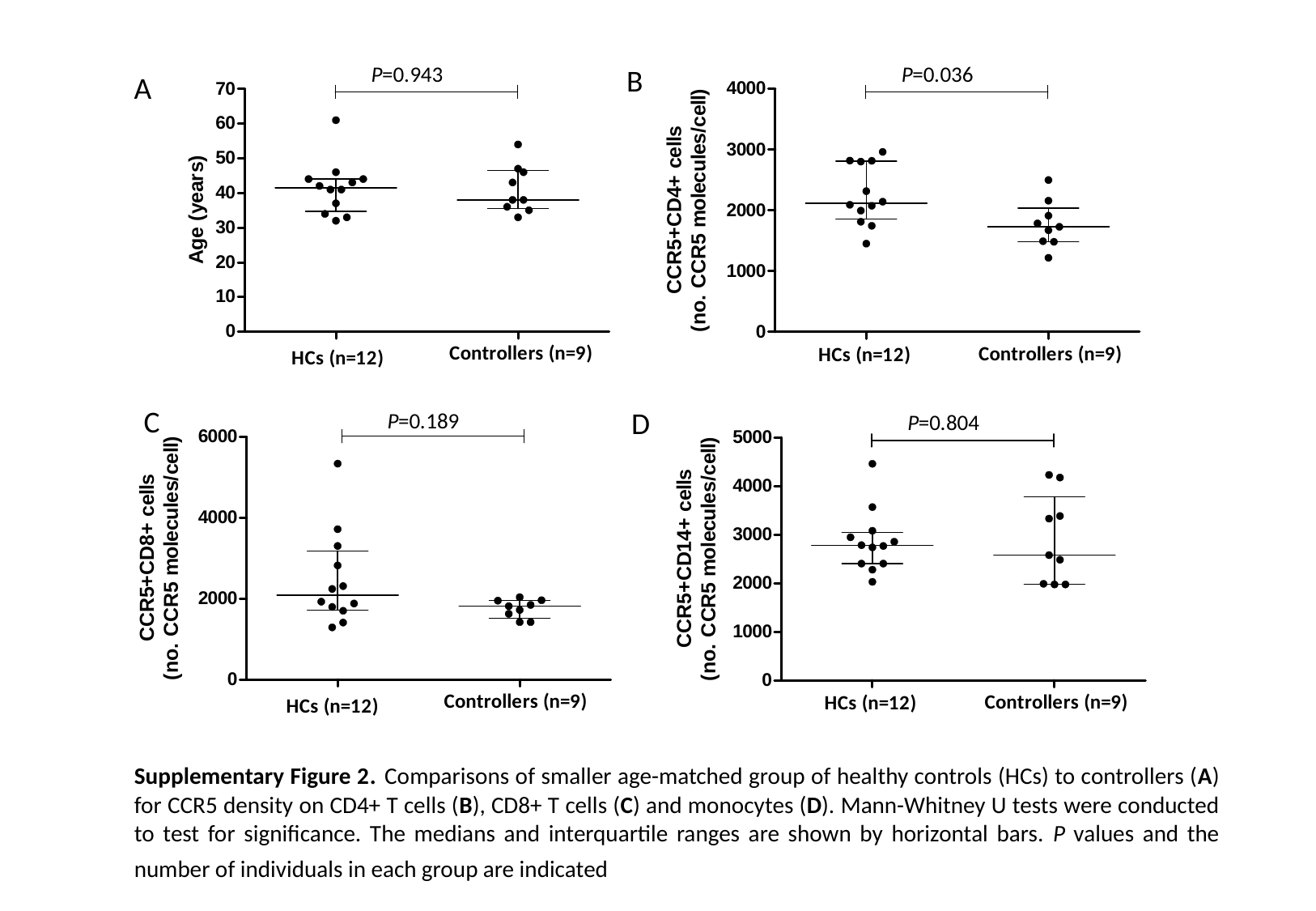

B
A
C
D
Supplementary Figure 2. Comparisons of smaller age-matched group of healthy controls (HCs) to controllers (A) for CCR5 density on CD4+ T cells (B), CD8+ T cells (C) and monocytes (D). Mann-Whitney U tests were conducted to test for significance. The medians and interquartile ranges are shown by horizontal bars. P values and the number of individuals in each group are indicated
