# Supplementary material for: Reduced CCR5 Expression and Immune Quiescence in Black South African HIV-1 Controllers
Source: Front Immunol. 2021 Dec 20;12:781263. doi: 10.3389/fimmu.2021.781263 (PMC8720782; doi:10.3389/fimmu.2021.781263)
Supplement: Supplementary file 3 [file Presentation_3.pptx]

## Slide 1
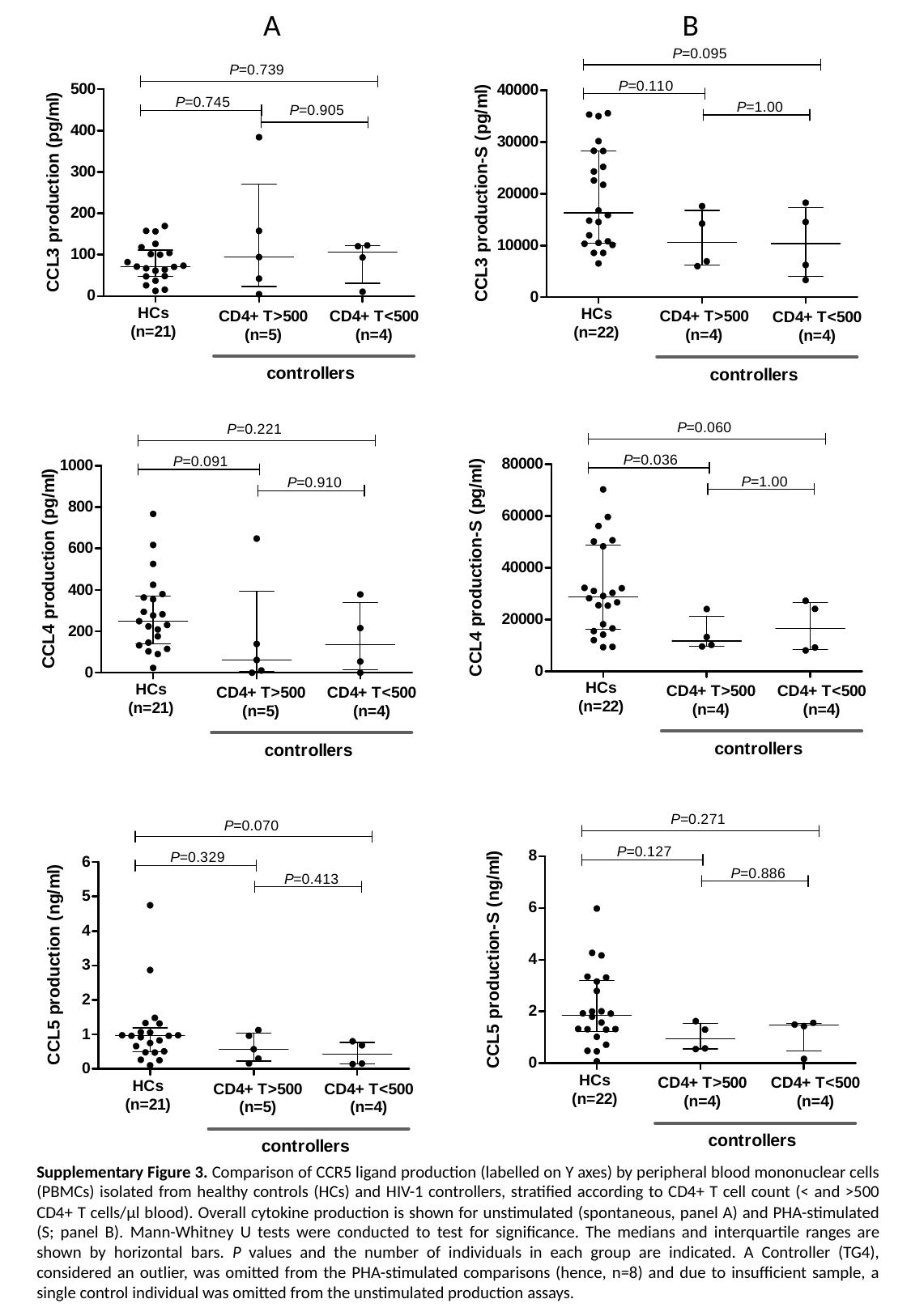

A
B
Supplementary Figure 3. Comparison of CCR5 ligand production (labelled on Y axes) by peripheral blood mononuclear cells (PBMCs) isolated from healthy controls (HCs) and HIV-1 controllers, stratified according to CD4+ T cell count (< and >500 CD4+ T cells/μl blood). Overall cytokine production is shown for unstimulated (spontaneous, panel A) and PHA-stimulated (S; panel B). Mann-Whitney U tests were conducted to test for significance. The medians and interquartile ranges are shown by horizontal bars. P values and the number of individuals in each group are indicated. A Controller (TG4), considered an outlier, was omitted from the PHA-stimulated comparisons (hence, n=8) and due to insufficient sample, a single control individual was omitted from the unstimulated production assays.
