# Supplementary material for: Reduced CCR5 Expression and Immune Quiescence in Black South African HIV-1 Controllers
Source: Front Immunol. 2021 Dec 20;12:781263. doi: 10.3389/fimmu.2021.781263 (PMC8720782; doi:10.3389/fimmu.2021.781263)
Supplement: Supplementary file 4 [file Presentation_4.pptx]

## Slide 1
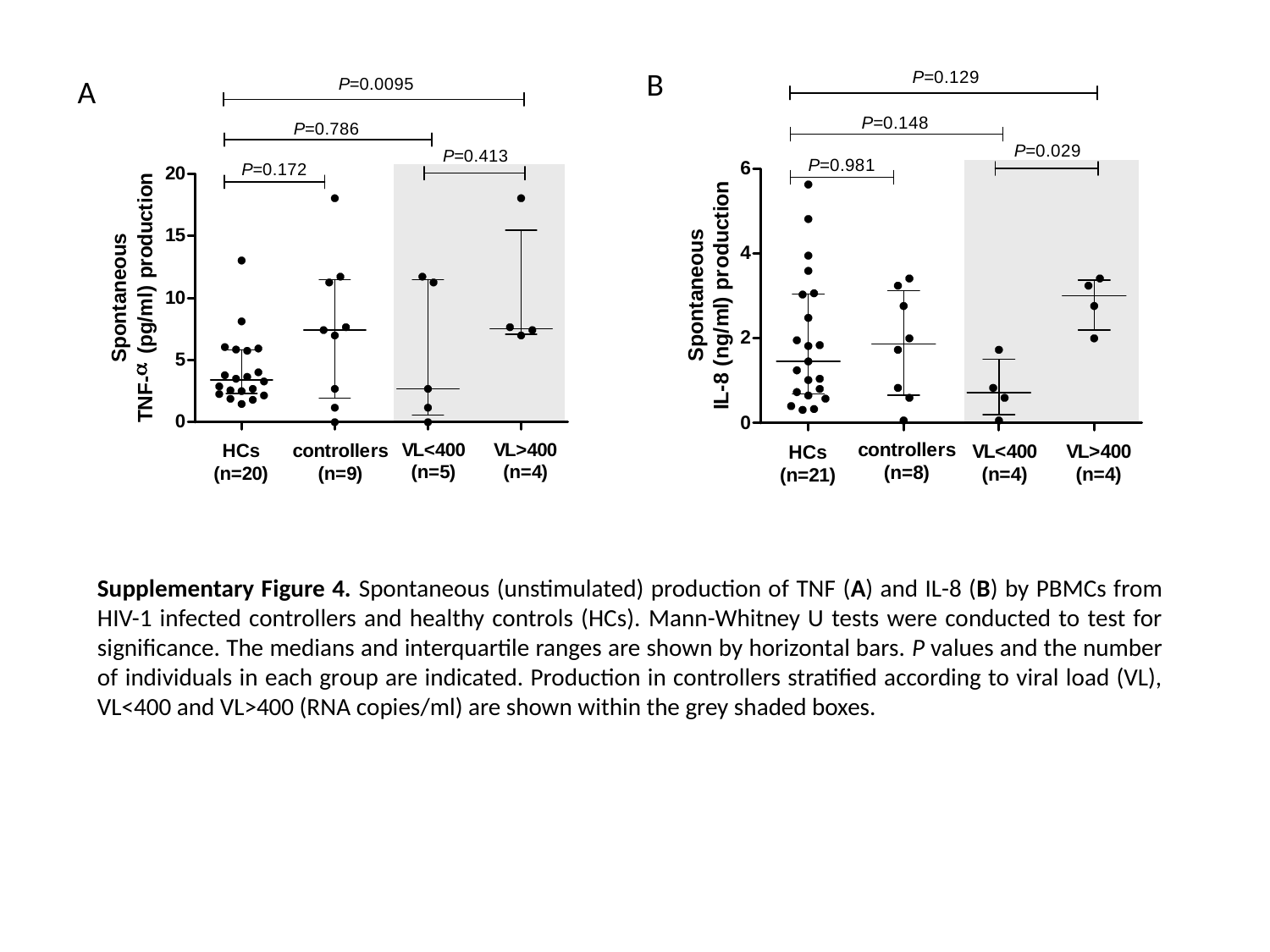

B
A
Supplementary Figure 4. Spontaneous (unstimulated) production of TNF (A) and IL-8 (B) by PBMCs from HIV-1 infected controllers and healthy controls (HCs). Mann-Whitney U tests were conducted to test for significance. The medians and interquartile ranges are shown by horizontal bars. P values and the number of individuals in each group are indicated. Production in controllers stratified according to viral load (VL), VL<400 and VL>400 (RNA copies/ml) are shown within the grey shaded boxes.
