# Supplementary material for: Reduced CCR5 Expression and Immune Quiescence in Black South African HIV-1 Controllers
Source: Front Immunol. 2021 Dec 20;12:781263. doi: 10.3389/fimmu.2021.781263 (PMC8720782; doi:10.3389/fimmu.2021.781263)
Supplement: Supplementary file 5 [file Presentation_5.pptx]

## Slide 1
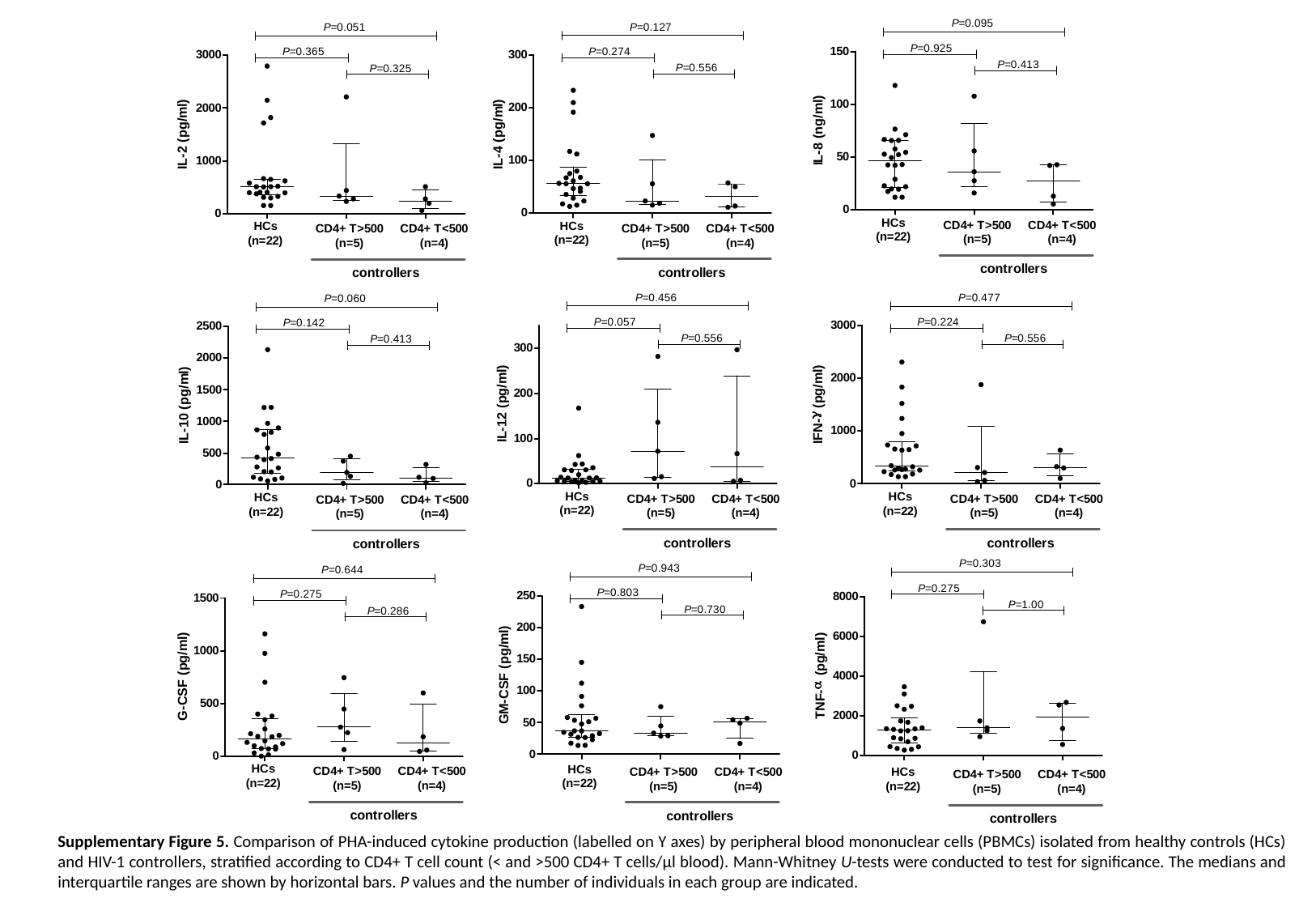

Supplementary Figure 5. Comparison of PHA-induced cytokine production (labelled on Y axes) by peripheral blood mononuclear cells (PBMCs) isolated from healthy controls (HCs) and HIV-1 controllers, stratified according to CD4+ T cell count (< and >500 CD4+ T cells/μl blood). Mann-Whitney U-tests were conducted to test for significance. The medians and interquartile ranges are shown by horizontal bars. P values and the number of individuals in each group are indicated.
