# Supplementary material for: Reduced CCR5 Expression and Immune Quiescence in Black South African HIV-1 Controllers
Source: Front Immunol. 2021 Dec 20;12:781263. doi: 10.3389/fimmu.2021.781263 (PMC8720782; doi:10.3389/fimmu.2021.781263)
Supplement: Supplementary file 6 [file Table_1.docx]

Supplementary Table 1. Details of individuals in healthy control cohort

| **Patient ID** | **Age**  **(years)** | **Gender** | ***CCR5* genotype** |
| --- | --- | --- | --- |
|  |  |  |  |
| CCR22 | 32 | F | HHC/ HHD |
| CCR24 | 29 | M | HHA/ HHE |
| CCR25 | 33 | M | HHC/ HHD |
| CCR26 | 41 | F | HHA/ HHF*2 |
| CCR27 | 61 | M | HHD/ HHD |
| CCR29 | 29 | F | HHD/ HHE |
| CCR30 | 27 | F | HHC/ HHE |
| CCR31 | 29 | F | HHA/ HHE |
| CCR32 | 44 | F | HHA/ HHA |
| CCR33 | 44 | M | HHD/ HHE |
| CCR35 | 37 | F | HHA/ NEW |
| CCR37 | 43 | M | HHA/ HHC |
| CCR39 | 41 | F | HHF*2/ HHF*2 |
| CCR41 | 34 | F | HHA/ HHE |
| CCR42 | 46 | M | HHE/ HHE |
| CCR43 | 27 | F | HHC/ HHF*2 |
| CCR44 | 26 | F | HHA/ HHD |
| CCR45 | 26 | F | HHC/ HHF*2 |
| CCR46 | 29 | F | HHD/ HHD |
| CCR47 | 22 | M | HHC/ HHD |
| CCR49 | 28 | M | HHC/ HHE |
| NKNCD6 | 42 | F | HHD/ HHE |
