# Supplementary material for: Reduced CCR5 Expression and Immune Quiescence in Black South African HIV-1 Controllers
Source: Front Immunol. 2021 Dec 20;12:781263. doi: 10.3389/fimmu.2021.781263 (PMC8720782; doi:10.3389/fimmu.2021.781263)
Supplement: Supplementary file 7 [file Table_2.docx]

Supplementary Table 2. Characteristics of cohort 2 used for CCR5 mRNA analysis

| **Clinical Phenotype Groups** | **N** | **Age (years)**  **Mean and Range** | **Gender**  **% female** | **CD4+ T cell counts**  **(cells/μl blood)**  **Median and IQRs** | **Viral load**  **(HIV-1 RNA copies/ml plasma)**  **Median and IQRs** | **ART free time since HIV-1 diagnosis (years)^1^**  **Median and IQRs** |
| --- | --- | --- | --- | --- | --- | --- |
| Health controls (HCs-2) | 10 | 36  (23-58) | 70 | NA | NA | NA |
| HIV-1 controllers (controllers-2) | 20 | 39.85  (22-58) | 85 | 671  (541.3-875.5) | 743.5  (138.8-3713) | 12  (10.25-13.75) |
| HIV-1 progressors | 12 | 37  (20-55) | 58 | 140  (55-165) | 175 370  (30 500-771 185) | NA |

^1^: The time since diagnosis in the absence of antiretroviral therapy (ART) was calculated for 16/20 controllers since 4 controllers (1 elite controller and 3 viraemic controllers) had no information on date of first diagnosis and were enrolled based on enrolment viral load and CD4+ T cell count. Years since diagnosis are reported up to time-point of the sample used for the CCR5 mRNA analysis.
