# Supplementary material for: Reduced CCR5 Expression and Immune Quiescence in Black South African HIV-1 Controllers
Source: Front Immunol. 2021 Dec 20;12:781263. doi: 10.3389/fimmu.2021.781263 (PMC8720782; doi:10.3389/fimmu.2021.781263)
Supplement: Supplementary file 8 [file Table_3.docx]

Supplementary Table 3. Frequencies of identified polymorphisms within the black South African HIV-1 controllers and black background South African (SA) healthy population

| Location on gene | SNP Position | Base change (major allele/minor allele) | Accession number^a^ | controllers 2N=18  n (frequency^b^) | Background  SA  2N=82 |
| --- | --- | --- | --- | --- | --- |
|  | -5268 | G/A | rs3136535 | 1 (0.056) | 0.037 |
|  | -5080 | T/A | rs41429449 | 2 (0.111) | 0.049 |
|  | -5072 | C/T | rs35078594 | 3 (0.167) | 0.049 |
|  | -4745 | C/T | rs3136536 | 2 (0.111) | 0.122 |
|  | -4257 | A/C | rs41490645 | 1 (0.056) | 0 |
|  | -4223 | C/T | rs553615728 | 3 (0.167) | 0.049 |
|  | -4088 | T/C | rs41499550 | 1 (0.056) | 0.220 |
|  | -3899 | A/C | rs72622924 | 5 (0.278) | 0.171 |
|  | -3886 | C/T | rs41412948 | 1 (0.056) | 0 |
|  | -3868 | CTAT/- | rs10577983 | 5 (0.278) | 0.171 |
|  | -3458 | G/T | rs2734225 | 5 (0.278) | 0.159 |
|  | -3261 | G/A | rs41475349 | 1 (0.056) | 0.220 |
|  | -2852 | A/G | rs2227010 | 2 (0.111) | 0.244 |
| Exon 1  (57 bp) | -2733 | A/G | rs2856758 | 1 (0.056) | 0.043 |
| Intron 1  (501bp) | -2554 | G/T | rs2734648 | 6 (0.333) | 0.366 |
|  | -2459 | G/A | rs1799987 | 6 (0.333) | 0.402 |
| Exon 2A  (235bp) | -2135 | T/C | rs1799988 | 6 (0.333) | 0.402 |
|  | -2132 | C/T | rs41469351 | 1 (0.056) | 0.207 |
|  | -2086 | A/G | rs1800023 | 5 (0.278) | 0.159 |
| Intron 2  (1903bp) | -1835 | C/T | rs1800024 | 4 (0.222) | 0.159 |
|  | -1686 | A/C | rs9282632 | 5 (0.278) | 0.220 |
|  | -1464 | A/G | rs3181037 | 5 (0.278) | 0.220 |
|  | -1130 | AG/- | rs3054375 | 5 (0.278) | 0.159 |
|  | -1060 | C/T | rs2856762 | 1 (0.056) | 0 |
|  | -976 | C/T | rs2254089 | 5 (0.278) | 0.159 |
|  | -975 | G/A | rs41395249 | 2 (0.111) | 0.073 |
|  | -651 | C/T | rs2856764 | 5 (0.278) | 0.159 |
|  | -444 | G/A | rs2856765/  rs35046662 | 5 (0.278) | 0.159 |
|  | -362 | ACAA/G | rs71619644 | 5 (0.278) | 0.159 |
|  | -113 | G/T | rs3176763 | 5 (0.278) | 0.220 |
|  | -112 | G/A | rs41352147 | 1 (0.056) | 0.012 |
| 3' UTR  (2651bp) | +1752 | G/A | rs41495153 | 4 (0.222) | 0.244 |
|  | +1823 | C/T | rs17765882 | 1 (0.056) | 0 |
|  | +1843 | G/A | rs41418945 | 2 (0.111) | 0.110 |
|  | +1846 | G/A | rs41466044 | 2 (0.111) | 0.110 |
|  | +2066 | G/A | rs1701732807 | 1 (0.056) | 0.049 |
|  | +2077 | G/T | rs1800874 | 5 (0.278) | 0.146 |
|  | +2458 | A/C | rs3188094 | **5 (0.278)^c^** | **0.085** |
|  | +2772 | G insertion | NI | 1 (0.056) | 0.085 |
|  | +2919 | T/G | rs746492 | 7 (0.389) | 0.378 |

^a^Accession numbers of SNPs detected in this study which have been previously reported in the SNP database (dbSNP). NI indicates newly identified polymorphisms ([Picton *et al.* 2010](#_ENREF_68)).

^b^Frequency was calculated for both populations using total number of alleles, i.e., n=18 for controllers.

^c^Bold font indicates a significant difference (*P*<0.05).
