# Supplementary material for: Reduced CCR5 Expression and Immune Quiescence in Black South African HIV-1 Controllers
Source: Front Immunol. 2021 Dec 20;12:781263. doi: 10.3389/fimmu.2021.781263 (PMC8720782; doi:10.3389/fimmu.2021.781263)
Supplement: Supplementary file 9 [file Table_4.docx]

Supplementary Table 4. Genotype-matched comparison of CCR5 expression, both as percentage of CCR5-expressing cells and CCR5 density, between HIV-1 controllers and healthy controls (HCs) across all peripheral blood cell populations

| **Cell subset** | **Percentage of CCR5-expressing cells (%)** | | | | | **Mean number of CCR5 molecules per cell** | | | | |
| --- | --- | --- | --- | --- | --- | --- | --- | --- | --- | --- |
|  | **HCs (n=6)** | | **controllers (n=6)** | |  | **HCs (n=6)** | | **controllers (n=6)** | |  |
|  | median | range | median | range | ***P*** | median | range | median | range | ***P*** |
| **B cells** |  |  |  |  |  |  |  |  |  |  |
| CD19+CCR5+ | 23.5 | 21.0-40.7 | 31.4 | 7.85-90.6 | 0.792 | 1978 | 1326-5091 | 3651 | 1379-6886 | 0.352 |
| **T cells** |  |  |  |  |  |  |  |  |  |  |
| CD4+CCR5+ | 28.3 | 21.5-36.6 | 28.3 | 19.9-70.4 | 0.818 | 2324 | 1815-2822 | 1851 | 1223-2503 | 0.093* |
| CD8+CCR5+ | 56.7 | 42.9-71.1 | 61.8 | 42.3-84.3 | 0.699 | 2568 | 1413-4183 | 1834 | 1422-2035 | 0.240 |
| **NK cells** |  |  |  |  |  |  |  |  |  |  |
| CD56+CCR5+ | 26.4 | 7.52-64.5 | 34.3 | 13.9-49.0 | 0.937 | 1222 | 912-1641 | 1244 | 1118-1810 | 0.485 |
| CD56^dim^CCR5+ | 26.5 | 7.26-61.0 | 37.9 | 18.1-61.2 | 0.575 | 1221 | 976-1744 | 1033 | 701-2058 | 0.485 |
| CD56^bright^CCR5+ | 68.0 | 15.5-77.8 | 61.3 | 10.0-75.0 | 0.589 | 1011 | 702-1616 | 1406 | 1042-1810 | 0.093* |
| CD56+CD16+ | 26.4 | 6.02-52.4 | 29.2 | 13.9-52.2 | 0.818 | 1185 | 860-1751 | 1122 | 839-1483 | 0.699 |
| **Monocytes** |  |  |  |  |  |  |  |  |  |  |
| CD14+CCR5+ | 8.62 | 3.48-11.3 | 10.11 | 8.67-12.9 | 0.310 | 4716 | 3920-6371 | 3656 | 2792-4225 | 0.009 |

* Indicates a trend towards significance; grey shaded box indicates significant difference.
